# Diversity and assembly of root-associated microbiomes of rubber trees

**DOI:** 10.3389/fpls.2023.1136418

**Published:** 2023-03-31

**Authors:** Guoyu Lan, Yaqing Wei, Yuwu Li, Zhixiang Wu

**Affiliations:** ^1^ Rubber Research Institute, Chinese Academy of Tropical Agricultural Sciences, Haikou, Hainan, China; ^2^ Tropical Forestry Ecology Group, Hainan Danzhou Tropical Agro-ecosystem National Observation and Research Station, Danzhou, Hainan, China; ^3^ College of Ecology and Environment, Hainan University, Haikou, Hainan, China; ^4^ College of Landscape Architecture and Forestry, Qingdao Agricultural University, Qingdao, Shandong, China

**Keywords:** rubber tree, bacteria, fungi, rhizoplane, diversity, assembly

## Abstract

**Introduction:**

Understanding the diversity and assembly of the microbiomes of plant roots is crucial to manipulate them for sustainable ecosystem functioning. However, there are few reports about microbial communities at a continuous fine-scale of roots for rubber trees.

**Methods:**

We investigate the structure, diversity, and assembly of bacterial and fungal communities for the soil (non-rhizosphere), rhizosphere, and rhizoplane as well as root endosphere of rubber trees using the amplicon sequencing of 16S ribosomal ribonucleic acid (rRNA) and Internally Transcribed Spacer (ITS) genes.

**Results:**

We show that 18.69% of bacterial and 20.20% of fungal operational taxonomic units (OTUs) in the rhizoplane derived from the endosphere and 20.64% of bacterial and 20.60% of fungal OTUs from the soil. This suggests that the rhizoplane microbial community was a mixed community of soil and endosphere microbial communities and that microorganisms can disperse bidirectionally across different compartments of the plant root. On the other hand, in the absence of an enrichment or depletion of core bacterial and fungal OTUs in the rhizosphere, little differences in microbial composition as well as a more shared microbial network structure between the soil and the rhizosphere support the theory that the rhizosphere microbial community is a subset of the soil community. A large number of functional genes (such as nitrogen fixation and nitrite reduction) and more enriched core OTUs as well as a less stable but more complex network structure were observed in the rhizoplane of rubber tree roots. This demonstrated that the rhizoplane is the most active root compartment and a hotspot for plant–soil–environment interactions. In addition, bacterial and fungal communities in the rhizoplane were more stochastic compared to the rhizosphere and soil.

**Discussion:**

Our study expands our understanding of root-associated microbial community structure and function, which may provide the scientific basis for sustainable agriculture through biological process management.

## Introduction

The rhizosphere, which connects plants and soil, is home to a rich diversity of microorganisms, many of which profit plants by helping to acquire nutrients from the soil and suppressing the invasion of pathogens (Leach et al., 2017; Liu et al., 2021). Thus, the composition, diversity, and function of rhizosphere microorganisms are one of the most important and studied aspects in the field of microbial ecology (He et al., 2022; Ling et al., 2022). Understanding the taxonomic and functional components as well as the assembly of the root microbiome and how they differ from those of the soil microbiome is crucial to manipulate them for sustainable ecosystem functioning (Ling et al., 2022). The detailed structure, diversity, and assembly of root-associated microbial communities has been studied for different plants including rice (Edwards et al., 2015), mangrove (Zhuang et al., 2020), wheat, and faba bean (Attia et al., 2022). There are three continuous fine-scale compartments of the plant root that microbes occupy: rhizosphere, rhizoplane, and endosphere. Edwards et al. (2015) defined the three compartments as follows: the rhizosphere is the soil close to the root surface, the rhizoplane is the root surface, and the endosphere is root interior. Zhuang et al. (2020) suggested that a non-rhizosphere (bulk soil) can also represent the microhabitat of the plant root.

It is generally assumed that root-associated microorganisms mainly derive from soil (Ofek-Lalzar et al., 2014; Edwards et al., 2015). However, a great quantity of literature shows that there are significant differences in the composition between soil- and root-associated microorganisms due to the selection process (Bulgarelli et al., 2013; Peiffer et al., 2013; Edwards et al., 2015; Fan et al., 2017; Yan et al., 2017; Fan et al., 2018; Attia et al., 2022). Thus, both the soil environment and plants drive the root-associated microbial assembly (Bai et al., 2022). Soil properties, vegetation history (Barnett et al., 2020), and plant rhizodeposition can all affect microbial enrichment in the rhizosphere soil (Demoling et al., 2007; Dennis et al., 2010). The host plant genotype determines the production of exudates and the plant metabolome (Peiffer et al., 2013; Rotoni et al., 2022) and fine-tunes the composition of the rhizoplane and endosphere communities (Bodenhausen et al., 2014; Bulgarelli et al., 2015). Thus, plants assemble their microbiomes from not only the soil but also the endosphere, which is influenced by species-specific genetic factors (Bulgarelli et al., 2013).

The rubber tree is one of the most important economic crops in the tropics. Despite the considerable importance of natural rubber as a global commodity and the widespread cultivation of rubber trees across South-East Asia, little is known about the composition or diversity of its microbiome. Guo et al. (2013) analyzed the microbial community structure of the rubber tree by using phospholipid fatty acids. In our study, we investigate the composition, diversity, network, and assembly of microbiomes at a continuous fine-scale of roots of *Hevea brasiliensis* (rubber tree) by using 16S and ITS amplicon sequences. We aim to understand the compositional and functional differences of the microbial community in different compartments of the rubber tree root. We investigate the following hypotheses: (1) microorganisms can disperse bidirectionally at different compartments of rubber tree roots due to root-associated filters (Munoz-Ucros et al., 2021) and plant genetic factors fine-tuning on the microbial community (Bodenhausen et al., 2014; Bulgarelli et al., 2015), (2) the microbial community in the rhizosphere is a mixed community of soil and endosphere microbial communities, 3) a network structure of the microbial community in the rhizosphere is more complex but less stable compared to soil, (4) the assembling of the microbial community of plant roots varies between compartments. Our research results provide a theoretical basis for sustainable management for rubber plantations.

## Methods

### Study site

We selected Danzhou, Wanning, Ledong, Jinghong, Menglun, and Mengpeng, which are the major rubber plantation districts as our study sites in Hainan and Xishuangbanna (**Figure S1** and **Table S1**). From each site, we selected three plots that were equally distant from each other (every 5–15 km according to the actual situation). Thus, there were a total of six rubber tree plantation sites and 18 plots. We recorded the latitude, longitude, and elevation of each plot. The data for the mean annual precipitation and mean annual temperature were acquired from the National Meteorological Information Center (data.cma.cn) for further analysis in the following methods.

### Sampling

Three rubber trees, with a distance of approximately 100 m from each other, were selected in each plot as our study objectives. Top soil (0–20 cm) samples were collected from an unplanted area. In detail, soil samples were collected at the middle of two rubber trees and the center of three rubber trees (**Figure S2A**). After sieving, they were mixed to form a composite soil sample. Root samples were selected from the four directions (i.e., north, south, east, and west) of each rubber tree and then mixed to form a single composite sample (**Figure S2B**). Rhizosphere soil was manually separated from the roots by shaking, while leaving rhizoplane soil (i.e., ~1 mm thick layer of soil) still attached to the roots (Edwards et al., 2015; Richter-Heitmann et al., 2016; Attia et al., 2022). These roots were placed in sterile phosphate-buffered solution (PBS) and brought back to the laboratory for the isolation of the rhizocompartments as described below. To collect the rhizoplane suspensions, root segments were separately placed into microcentrifuge tubes containing 1 ml of PBS and shaken at 300 rpm for 15 min at 4°C. Next, the same root segments were washed three times in fresh PBS, transferred to new microcentrifuge tubes containing 1 ml of PBS, and then subjected to sonication using a sonication bath for 1 min at 4°C to collect the rhizoplane suspensions (Rp). Each rhizocompartment was isolated from the rubber tree sampled and had total DNA extracted. Soil- and root-associated microbiome sampling was performed twice: in July (rainy season) 2020 and January (dry season) 2021. Finally, we got 288 root-associated microbiome (bacteria and fungi) samples. We used the combined data of both dry season and rainy season to summarize the general rules of the diversity and assemblages for root-associated microbiomes.

Soil pH was measured in a 1:1 soil–water mixture. Soil moisture was measured gravimetrically. Soil total nitrogen (TN) was determined using a micro-Kjeldahl digestion followed by steam distillation. Total phosphorus (TP) and total potassium (TK) were digested with NaOH. Nitrate nitrogen (NN) and ammonium nitrogen (AN) were determined by steam distillation and indophenol-blue colorimetry, respectively. Soil samples were extracted with NaHCO_3_, and the extracts were then used to measure the available soil phosphorus (AP) using molybdate-blue colorimetry. For soil potassium (AK), soil samples were first extracted with ammonium acetate before loading the extracts onto an atomic absorption spectrometer with ascorbic acid as a reductant (Chen et al., 2019).

### DNA extraction and PCR amplification

Microbial DNA was extracted from 0.5 g of soil using the E.Z.N.A.^®^ Soil DNA Kit (Omega Bio-tek, Norcross, GA, USA) following the manufacturer’s protocol. The fungal ITS1 hypervariable region was amplified using the PCR primers ITS1F (5’-CTTGGTCATTTAGAGGAAGTAA-3’) and ITS2R (5’-GCTGCGTTCTTCATCGATGC-3’) (Adams et al., 2013). For bacteria and archaea, the V4 hypervariable region of the bacterial 16S rRNA gene was amplified using the PCR primers 515FmodF (5’-GTGYCAGCMGCCGCGGTAA-3’) and 806RmodR (5’-GGACTACNVGGGTWTCTAAT-3’) (Sampson et al., 2016; Walters et al., 2016). The PCR reactions were conducted using the following approach: an initial 3 min denaturation at 95°C, followed by 27 cycles of 30 s at 95°C, 30 s of annealing at 55°C, and 45 s of elongation at 72°C and a 10 min final extension at 72°C. Purified amplicons were combined equimolarly, and paired-end sequencing (2 × 250) was performed on the Illumina MiSeq platform at Shanghai Majorbio Bio-pharm Biotechnology Co., Ltd. (Shanghai, China) according to standard protocols. The raw reads were deposited into the National Center for Biotechnology Information Sequence Read Archive database (Accession Number: SRP342019).

### Bioinformatics and data analysis

Raw fastq files were demultiplexed and quality-filtered using Quantitative Insights Into Microbial Ecology (QIIME) (Caporaso et al., 2010) (version 1.17). During filtering, the sequences were trimmed with a moving window of 50 bp and a quality threshold score of 30. The dataset was then simplified by eliminating singletons (Zhuang et al., 2020). Operational taxonomic units (OTUs) were clustered with a 97% similarity cutoff using UPARSE (Edgar, 2013) (version 7.1 http://drive5.com/uparse/), and chimeric sequences were identified and removed using UCHIME. Using the RDP Classifier (http://rdp.cme.msu.edu/), the phylogenetic affiliation of each 16S rRNA gene and ITS gene sequence was determined using a confidence threshold of 70% with the SILVA 16S rRNA database and UNITE database, respectively (Quast et al., 2013; Nilsson et al., 2019). The relative abundance was determined for each taxa (Good, 1953), and the diversity indices were calculated based on resampled sequence data using MOTHUR (http://www.mothur.org) (Schloss et al., 2009).

### Data analysis

We evaluated the diversity of the root-associated bacterial and fungal community of four compartments of rubber trees. Community α-diversity was estimated by using the Chao1 index. Differences in the bacterial and fungal community composition were visualized by principal coordinates analysis (PCoA) based on Bray–Curtis distance matrices and further tested by the analysis of similarities (ANOSIM) using the ANOSIM functions in the “vegan” package (Oksanen et al., 2022) in the R environment (R Core Team, 2022). Community β-diversity was estimated using a permutational analysis of multivariate dispersions (PERMDISP). Compartment differences were assessed by comparing the average distance to the centroid in the PERMDISP analysis. PERMANOVA was also performed to test the differences of the four compartments. Cumulative numbers of OTUs for all samples of each compartment of the rubber tree root were used to estimate the γ-diversity.

A generalized liner model approach was applied for detecting significantly (*p*-value < 0.05) enriched and depleted OTUs in the bacterial and fungal communities of a specific compartment compared to those of soil (Edwards et al., 2015; He et al., 2022). Fold change (FC) was defined as the ratio of the microbial abundance of a specific compartment to that of soil. Those with FC greater than 1 and with *p* < 0.05 are considered as enriched OTUs, while FC lower than 1 with *p* < 0.05 are considered as depleted OTUs. Then, the enriched and depleted pattern of each compartment compared with soil were illustrated with a volcano map by using ggplot2 packages in the R environment. Core bacterial OTUs were defined as follows: (i) present in all samples of each compartment and (ii) with a relative abundance (RA) > 0.01% (He et al., 2022). For fungi, we defined those present in at least 60% of samples of each compartment and with a relative abundance ≥ 0.01% as core OTUs (Xiong et al., 2021). Finally, the phylogenetic tree of all core bacterial and fungal OTUs for each compartment was conducted on the free online platform of Majorbio I-Sanger Cloud Platform (www.i-sanger.com), and the tree was displayed using Interactive Tree of Life (ITOL) (Letunic and Bork, 2011).

We use Fast Expectation-maximization microbial Source Tracking (*FEAST*) to unravel the origins of complex microbial communities (Shenhav et al., 2019). FEAST was performed by using the package “FEAST” in the R environment. Functional Annotation of Prokaryotic Taxa (FAPROTAX) and Funguild were used for the prediction of bacterial (Louca et al., 2016) and fungal (Nguyen et al., 2016) functions of the four compartments of the rubber tree root, respectively.

The top 500 most abundant bacterial and fungal OTUs as well as core bacterial and fungal OTUs were used to analyze the network structure of four compartments. The top 500 OTUs roughly match the OTUs with a relative abundance greater than 0.05% (Jiao et al., 2016; Lan et al., 2022b). Four networks, representing each compartment (soil, rhizosphere, rhizoplane, and root endosphere) were constructed with 36 samples each. Interactions consisted of Spearman’s rank correlations. Co-occurrence networks were constructed using only significant correlations of ρ > 0.6 (*P* < 0.01) (Barberan et al., 2012) as this cutoff includes a range of interaction strengths (De Vries et al., 2018). Modularity and degree were calculated by using igraph package (Csardi and Nepusz, 2006) in the R environment, and the networks were then visualized in the Gephi environment (Bastian et al., 2009). The number of shared edges and unique edges of two compartments were calculated to evaluate how these network structures change from the soil to the endosphere.

The normalized stochasticity ratio (NST, based on Jaccard dissimilarity) was used to quantitatively evaluate whether community assembly was more deterministic (<50%) or stochastic (>50%) (Ning et al., 2019, Lan et al., 2022a). The NST was calculated by using the “NST” package in the R environment. Then, “nst.panova” was used to test whether the difference among the four compartments is significant. The NST for the microbial community was visualized by using the “ggplot2” package (Wickham, 2016) in the R environment.

## Results

### Community composition

**Figure 1:**
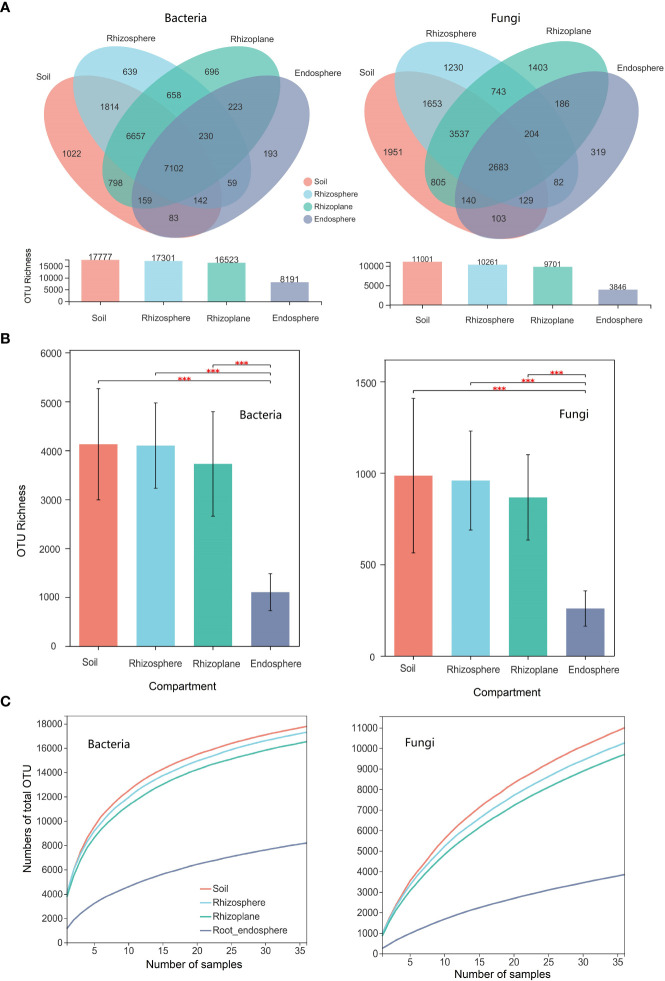
Microbial diversity of different compartments (soil, rhizosphere, rhizoplane, and root endosphere) of rubber tree root. **(A)**: Venn diagram depicting the shared and specific number of operational taxonomic units (OTUs) across compartments; **(B)**: Bar plot showing the OTU richness of microbial communities of different compartments; **(C)**: Cumulative numbers of OTUs for all samples (γ-diversity) of different compartments of the rubber tree root. *** *p* < 0.001.

There were a total of 1,777, 17,301, 16,523, and 8,191 bacterial OTUs in the soil, rhizosphere, rhizoplane, and endosphere, respectively. The number of unique bacterial OTUs (i.e., only existing in one compartment) in the soil, rhizosphere, rhizoplane, and endosphere is 1022, 639, 696, and 193, respectively (**Figure 1A**). The number of shared bacterial OTUs between the soil and the rhizosphere is 15,715, while the number of shared bacterial OTUs between the soil and the rhizoplane is 14,716. For bacterial composition, the relative abundance of Proteobacteria was significantly higher in the rhizoplane and endosphere than those of other compartments (*p* = 0.001), whereas the relative abundance of Chloroflexi in the rhizoplane ranks the lowest in the four compartments (*p* = 0.001) (**Figure S3**).

There were a total of 11,001, 10,261, 9,701, and 3,846 fungal OTUs in the soil, rhizosphere, rhizoplane, and endosphere, respectively. The number of unique fungal OTUs (i.e., only existing in one compartment) in the soil, rhizosphere, rhizoplane, and endosphere is 1,951, 1,230, 1,403, and 319, respectively (**Figure 1A**). The number of shared fungal OTUs between the soil and the rhizosphere is 8,002, while the number of shared bacterial OTUs between the soil and the rhizoplane is 7,165. For fungal composition, the relative abundance of Ascomycota and Glomeromycota ranks first in the root endosphere (*p* = 0.001) and Basidiomycota ranks the first in the rhizosphere (*p* = 0.05).

The PCoA ordination shows that the samples of the root endosphere are distributed on one side of the panel, whereas other samples are on the other side of the panel (**Figure S4**), indicating that microbial composition in the root endosphere is totally different from that in other compartments, which were further confirmed by the ANOSIM (**Table S2**). However, there were no significant difference in bacterial (*R* = 0138, *P* = 0.7840) and fungal (*R* = 0.226, *P* = 0.987) communities between the soil and the rhizosphere (**Table S2**)

### Core bacteria and fungi

**Figure 2:**
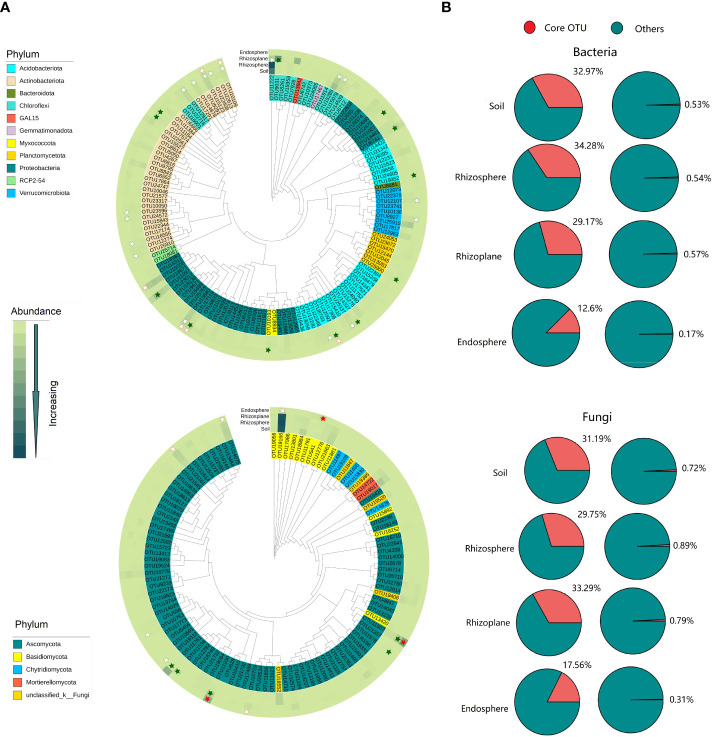
Composition and phylogenetic tree of core microbes in different compartments of the root of rubber trees. **(A)**: Phylogenetic tree of core bacterial and fungal communities in different compartments. Solid star represents enriched OTUs, open star represents depleted OTUs, red indicates enriched or depleted OTUs in the endosphere, and green indicates enriched or depleted OTUs in the rhizoplane. **(B)**: The percentage of core bacteria and fungi in abundance and OTU richness. Core bacterial OTUs: present in all samples of each compartment and with a relative abundance >0.01%. Core fungal OTUs: present in at least 60% samples of each compartment and with a relative abundance ≥0.01%.

More enriched or depleted core bacterial and fungal OTUs were observed in the rhizoplane. However, no enriched or depleted core bacterial and fungal OTUs was observed in the rhizosphere (**Figure 2** and **Table S3**). A total of 94, 99, 90, and 14 bacterial OTUs were defined as core OTUs in the soil, rhizosphere, rhizoplane, and endosphere, respectively, accounting for 0.17%–0.53% of the total OTU richness, but 12.6%–34.28% of the total number of sequences (**Figure 2** and **Figure S5**). These OTUs mainly belong to Proteobacteria, Acidobacteria, Actinobacteria, and Chloroflex. Similarity, a total 79, 91, 77, and 12 fungal OTUs were defined as core OTUs in soil, rhizosphere, rhizoplane, and endosphere respectively. These OTUs mainly belong to Ascomycata and Basidiomycota (**Figure S6**).

### Enriched and depleted operational taxonomic units

**Figure 3:**
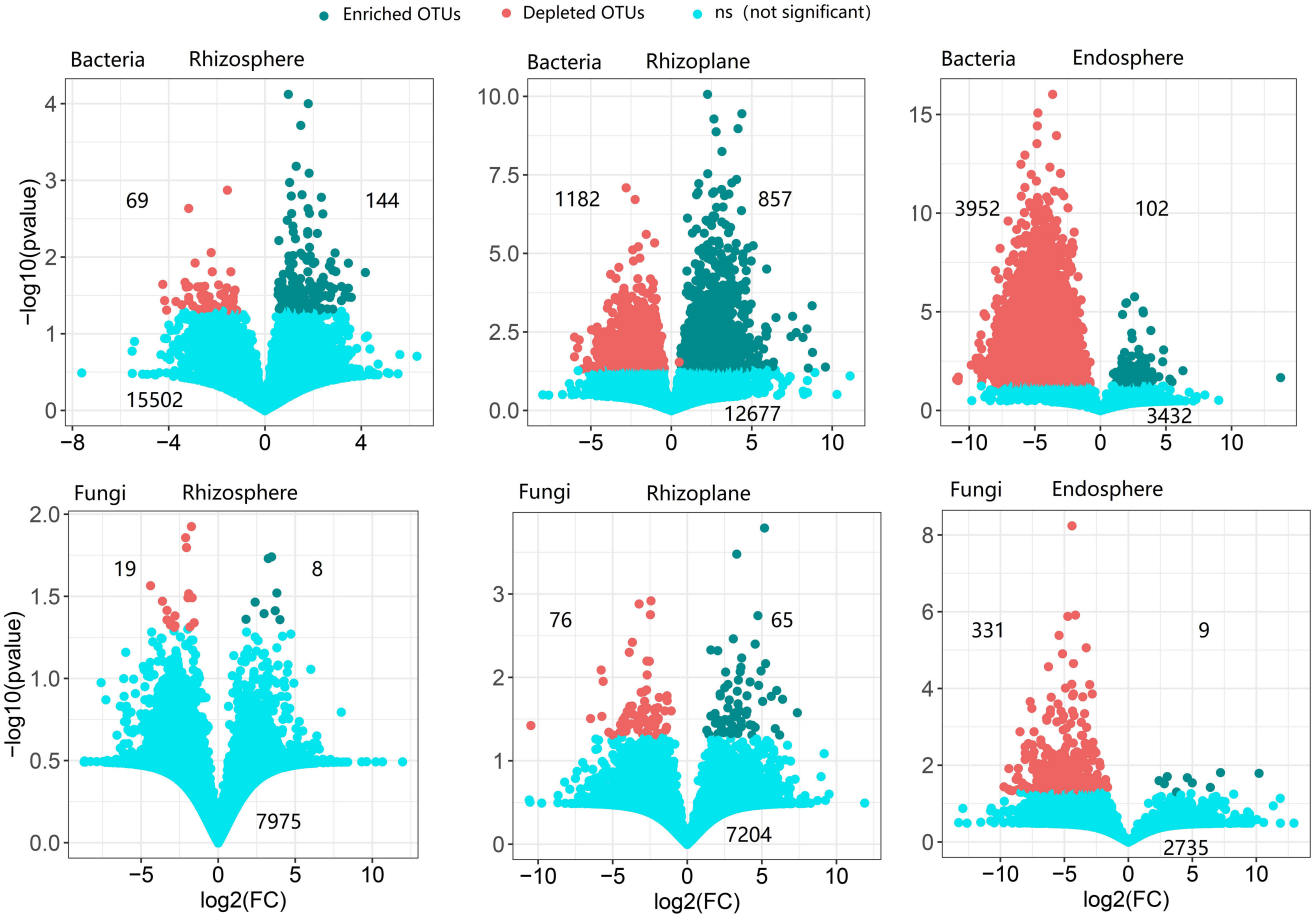
The enrichment and depletion patterns of the root-associated bacterial and fungal microbiomes in each compartment compared with soil. Each green point represents an individual enriched OTU, and a red point represents an individual depleted OTU. The x-axis represents the fold change (FC) in the abundance of a specific compartment compared with soil, and the y-axis reports the negative value of the logarithm (base10) of the *p*-value. Numbers on the top left of the panel indicate depleted OTUs, and numbers on the top right of the panel indicate enriched OTUs, while numbers on the bottom of the panel indicate not-significant OTUs.

The rhizoplane possessed more enriched bacterial and fungal OTUs (for bacteria, 857 OTUs, and for fungi, 65 OTUs) than rhizosphere (114 bacterial OTUs and 8 fungal OTUs) and endosphere (102 bacterial OTUs and 9 fungal OTUs) (**Figure 3**). Enriched bacterial OTUs mainly belong to Proteobacteria, Actinobacteria, Chloroflex, and Ascomycota (**Figure S7**). On the contrary, the endosphere possessed more depleted OTUs (3,952 bacterial OTUs and 331 fungal OTUs) than the rhizosphere (69 bacterial OTUs and 19 fungal OTUs) and rhizoplane (1,182 bacterial OTUs and 76 fungal OTUs). This suggests that the rhizoplane acts as an important gate for filtering microbes entering the inside of root. More importantly, the enriched and depleted core OTUs display relatively close clustering within the phylogenetic tree (**Figure 2**). The enriched OTUs in the core bacterial community of rhizoplane mainly belong to Proteobacteria, Acidobacteria, and Actinobacteria (**Figure 2**, **S6** and **Table S3**).

### Microbe source tracking

**Figure 4:**
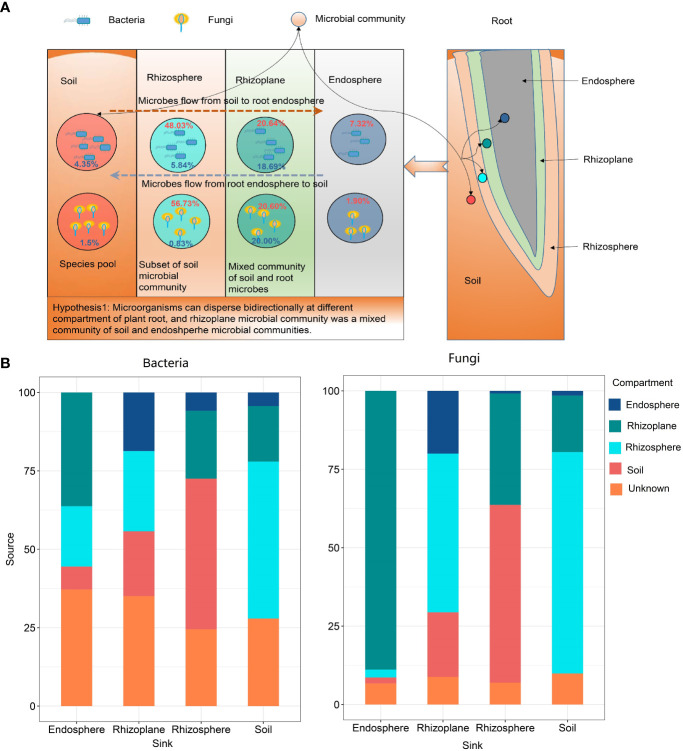
The potential sources of rubber tree root–associated bacterial and fungal communities of different compartments estimated by FEAST. **(A)**: The schematic diagram showing the bidirectional flow of microbes. Red numbers in circle indicate the percentage of microbes from soil, while blue numbers indicate microbes from the root endosphere; **(B)**: Bar plot illustrating the potential source of microbial communities in different compartments.

The FEAST results showed that rubber root–associated bacterial and fungal communities were partly derived from soil and gradually filtered in different compartments (**Figure 4** and **Table S4**). On the other hand, approximately 20.0% of the fungal OTUs and 18.69% of the bacterial OTUs in the rhizoplane came from the root endosphere, which indicates that the microorganisms in the root endosphere can also penetrate the root surface, thus affecting the composition of the microbial community on the root surface.

### Microbial gene functions

**Figure 5:**
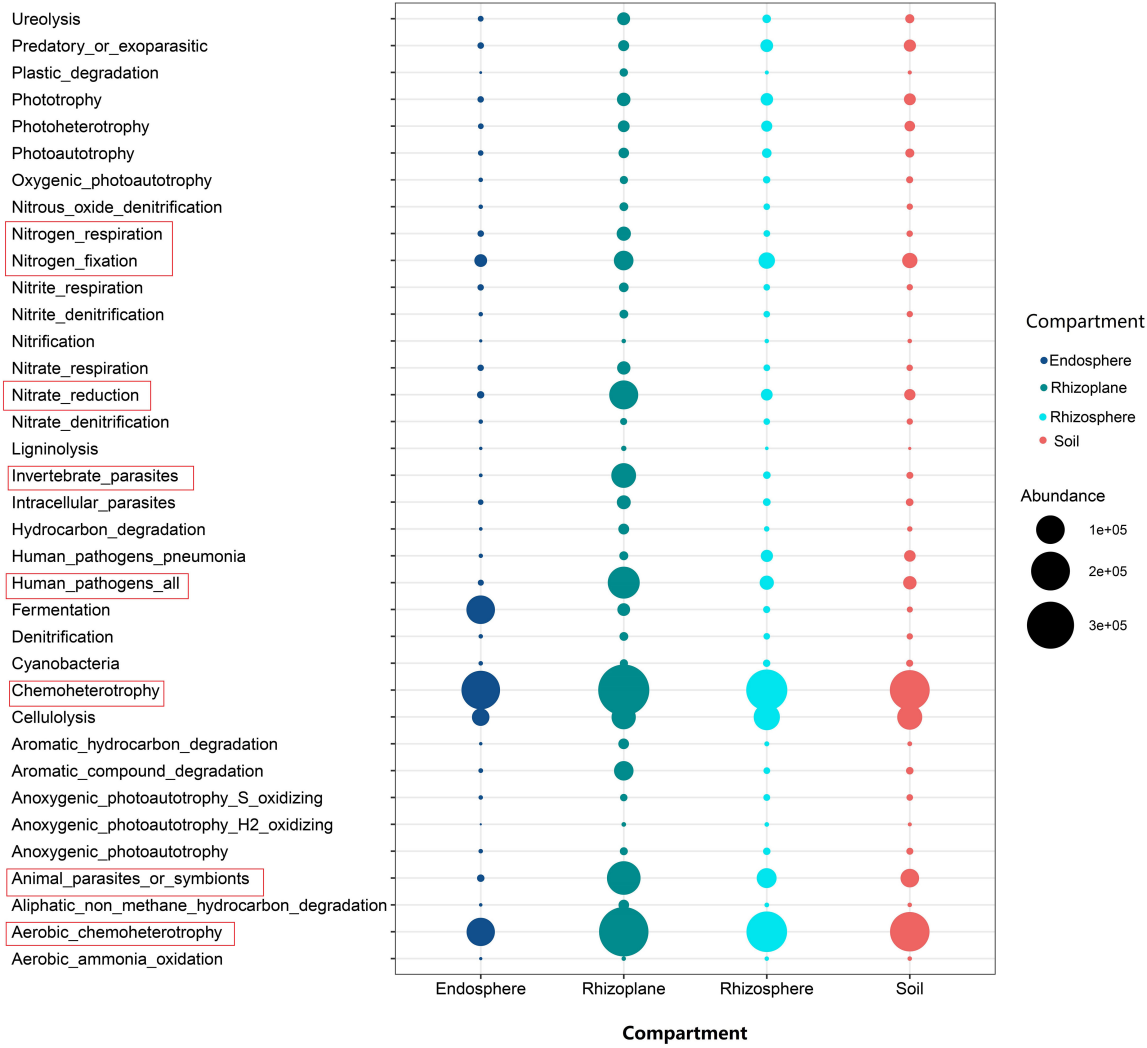
The bubble chart showing the abundance of functional gene of bacteria inferred by Functional Annotation of Prokaryotic Taxa for different compartments of the rubber tree root.

A large number of functional genes, such as nitrogen fixation, nitrite reduction, nitrogen respiration, and chemoheterotrophy, were enriched in the root rhizoplane, indicating that the rhizoplane is the most active root compartment for microorganisms (**Figure 5**). For fungal groups, the relative abundance of arbuscular mycorrhizal fungi in the root endosphere was relatively higher than those of other compartments, whereas the relative abundance of saprotrophs were lower in the root endosphere than in the rhizosphere, rhizoplane and soil (**Figure S8**).

### Community diversity

Here, we use the Chao1 index to estimate the α-diversity of the four compartments of the root. The Chao1 index of the OTU level for bacterial and fungal communities in the root endosphere was notably lower than those in other three compartments. However, there were no significant differences among the observed OTU richness of the rhizoplane, rhizosphere, and soil (**Figure 1B**). The beta diversity of the bacterial and fungal community (i.e., variation) was notably higher in the root endosphere than that in the soil, rhizosphere, and rhizoplane (*p* < 0.001) (**Figure S4**), indicating that the endosphere environment drove divergence in bacterial and fungal community composition. The number of cumulative OTUs for all samples were used to estimate the γ-diversity of the microbial community for different compartments of the root for the rubber tree (**Figure 1C**). Soil had the highest γ-diversity, followed by the rhizosphere and rhizoplane, and the root endosphere has the lowest γ-diversity (see also **Figure 1A**).

### Community network

**Figure 6:**
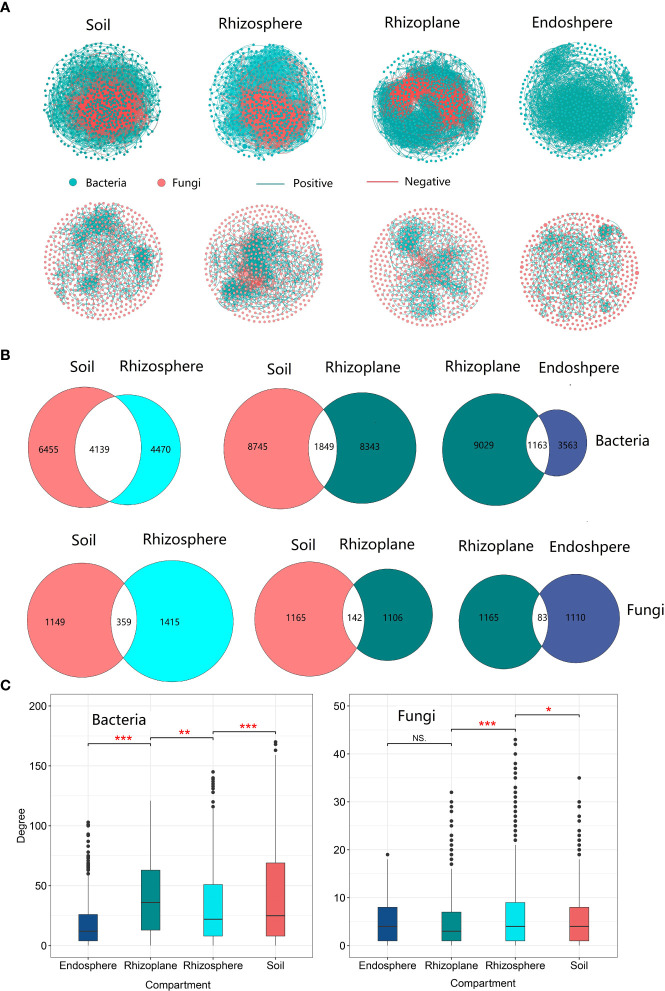
The microbial community network structure of different compartments of rubber tree root. **(A)**: Network of bacterial and fungal communities of different compartments, Green solid circles indicate bacteria; red solid circles indicate fungi. Red lines indicate negative correlation between OTUs, and green lines indicate positive correlations; **(B)** Number of shared and unique edges of soil bacterial and fungal networks in different compartments. The number where the two circles cross is the number of shared edges; **(C)** Mean degree of the network for the microbial communities of different compartments. * p < 0.05, ** p < 0.01, *** p < 0.001.

The network in soil had the largest number of edges compared to other compartments, and there were a total of 10,594 and 1,508 edges for soil bacterial and fungal networks, respectively (**Table S5**). The proportion of shared edges (4,139 for bacteria and 359 for fungi) between the soil and rhizosphere microbial communities was relatively higher compared to the proportion (1,849 for bacteria and 142 for fungi) between the soil and the rhizoplane. The proportion of shared edges reached 48.08% in the bacterial community, indicating that the rhizosphere bacterial community network is a subset of the soil network. However, the proportion (21.64%) of shared edges between the rhizosphere and soil network structure is relatively low (**Figure 6B**), indicating that the rhizoplane bacterial network is not a subset of the soil microbial network. The network degree of the rhizoplane bacterial community was the highest among the four compartments (**Figure 6C** and **Table S5**), while the highest degree of the fungal network was observed in the rhizosphere. Compared with the rhizosphere, the network of the rhizoplane bacterial community was the most complex but the least stable due to more edges, but less negative edges were observed in the rhizoplane. The network of core bacterial and fungal communities also revealed that the rhizoplane was the most complex among the three compartments (**Figures S9–S11** and **Table S6**).The network of core bacterial and fungal communities also revealed that the rhizoplane was the most complex among the three compartments (**Figures S9–S11** and **Table S6**).

### Community assembly

**Figure 7:**
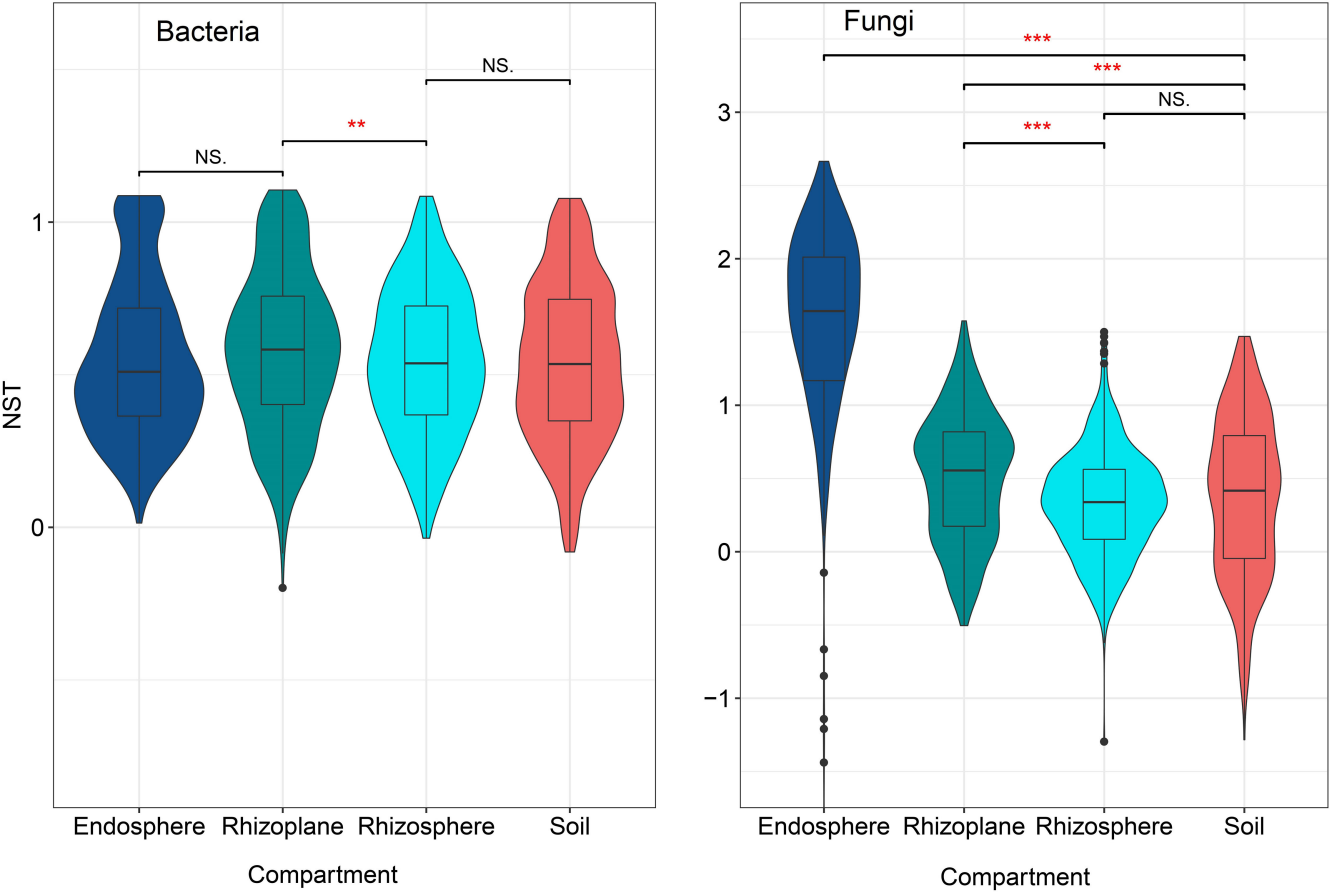
The normalized stochasticity ratio (NST) for bacterial and fungal communities in different compartments of the rubber tree root. NST < 50%: community assembly is more deterministic; NST > 50%: community assembly is more stochastic. ** p < 0.01, *** p < 0.001, NS, not significant.

The NST index of the bacterial community in the rhizoplane was the lowest among the four compartments (*p* < 0.05) (**Figure 7**) suggesting that the bacterial community of the rhizoplane was more stochastic than other compartments. However, for the fungal community, the NST index of the endosphere was the highest and then followed by the rhizoplane, soil, and rhizosphere, indicating that the fungal community of endosphere was more stochastic than those of other compartments of the root. Both bacterial and fungal communities in the rhizoplane were more stochastic than those in the rhizosphere.

## Discussion

### Rhizoplane microbial communities are a mixed community for soil and endosphere

It is generally believed that root-associated microorganisms come from soil (Ofek-Lalzar et al., 2014). Our findings demonstrated a decreasing gradient of γ-diversity from soil to the root endosphere, which was consistent with Edwards et al. (2015) reports that the soil community had higher γ-diversity than the rhizosphere. This can be easily understandable that the rhizosphere or rhizoplane was a more homogeneous environment compared to soil (Li et al., 2021), which will result in a lower γ-diversity. The decreased α-diversity with root proximity was inferred to the result of the root “filtration effect” (Dibbern et al., 2014). It was well known that the rhizoplane was primarily responsible for controlling the entry of specific microbial populations into the root (Durán et al., 2018), resulting in the selective enrichment of Proteobacteria and Acidobacteria in the endosphere (**Figure S7**). We also detected that the relative abundances of Acidobacteria and Gemmatimonadetes decrease from the soil to the root endosphere (**Figure S3**), which is consistent with previous studies for other plants (Bulgarelli et al., 2012; Lundberg et al., 2012; Schlaeppi et al., 2014; Edwards et al., 2015). These further verified the theory that host plants exert selective effects on the soil microbiota (Walker et al., 2003).

Microbial source track results showed that 88.87% fungal OTUs and 36.28% bacterial OTUs in the endosphere derived from the rhizoplane, indicating that microorganisms can disperse from the rhizoplane to root endosphere. However, there is still a lack of evidence that microorganisms in roots can penetrate the root surface and colonize in the rhizoplane or rhizosphere. Microbial source track results revealed that approximately 20% of microorganisms can penetrate the root surface and colonize in the rhizoplane. This confirmed the hypothesis that microorganisms can disperse bidirectionally across different compartments of the plant root. Thus, we suggest that microbial communities in the rhizoplane are a mixed community of those in the soil and endosphere.

### Rhizosphere microbial community is a subset of soil microbial community.

A large quantity of literatures demonstrated that the soil had higher α-diversity (Attia et al., 2022; Ceja-Navarro et al., 2021; Li et al., 2021; Ling et al., 2022). However, a study about *Phragmites australis* showed that the rhizosphere has higher α-diversity than that of the soil (He et al., 2022), which can be explained by the fact that the rhizosphere provides more suitable living conditions for bacteria than the soil, such as higher nutrient availability and weaker environmental stress. Our finding demonstrated that there was no significant difference in the microbial α-diversity between the soil and the rhizosphere. We also detected no significant difference in bacterial and fungal composition between the soil and the rhizosphere (**Table S2**). In addition, no core bacterial and fungal core OTUs were enriched in the rhizosphere compared to the soil (**Table S3**). These findings suggest that the rhizosphere microbial community is a subset of the soil microbial community.

In addition, our result revealed that rhizosphere bacterial co-occurrence networks were less complex than the soil networks (**Figure 6A** and **Table S5**), which was consistent with a previous study on wheat (Fan et al., 2017) but is not in line with previous study on *Bothriochloa ischaemum* (Wang et al., 2022). There are two reasons for the less complex microbial community network in the rhizosphere. Firstly, prolonged phytoextraction in the rhizosphere could decrease microbial network complexity (Luo et al., 2021). Secondly, the relative higher percentage of shared edges by the rhizosphere and soil microbial community (**Figure 5**), and no enriched and depleted core microbial OTUs in the rhizosphere (**Figure 2** and **Table S3**), suggested that the rhizosphere community is a subset of the soil community (Mendes et al., 2014). Thus, a less complex network in the rhizosphere is expected. Combined with previous research, which demonstrated different pioneer plant species having similar rhizosphere microbial communities (Ye et al., 2021), our findings may possibly support the theory that the rhizosphere community is a subset of the soil community (Mendes et al., 2014).

### Rhizoplane is the most active root compartment, with more complex but less stable network structure

Our finding conformed the universal law that endosphere communities had the lowest α-diversity (see **Figure 1**) among all compartments of the root (Edwards et al., 2015; Zhuang et al., 2020). At present, no clear consensus has been reached on whether the rhizosphere microbial community has higher diversity than the rhizoplane community. Edwards et al. (2015) reported that the rhizosphere had higher diversity than the rhizoplane, while the Zhuang et al. (2020) study suggested that the rhizoplane had higher bacterial and fungal α-diversity than that of the rhizosphere. Attia et al. (2022) suggests that the rhizoplane communities are a random subset of the rhizosphere communities. However, microbial source track revealed that 18.69% of bacteria and 20.00% of fungi in the rhizoplane derived from the endosphere, which indicates that the rhizoplane microbial community is affected by both plant genetic factors and the soil and a mixed community of the soil and rhizosphere microbial communities.

Our results showed that the abundance of genes related to nitrification was higher in the soil than that in rhizocompartments (**Figure 5**), which is consistent with the previous report on the maize root (Ai et al., 2013; Bay et al., 2021). Nitrification is prone to take place in aerobic conditions, but the rhizosphere generally suffers from oxygen deficiency (Lecomte et al., 2018) because roots and microorganisms consume more oxygen than the bulk soil (Ling et al., 2022). In addition, our results demonstrated that the relative abundance of genes related to nitrate reduction was higher in the rhizoplane than in the rhizosphere and soil. This is consistent with a previous study demonstrating that the functions responsible for nitrification are depleted in rhizocompartments (Ling et al., 2022). In fact, nitrate reduction can eliminate the toxic effect of nitric acid accumulation on plants (Zsoldos et al., 1993). In general, a large number of functional genes, such as nitrogen fixation and nitrite reduction, were enriched in the root rhizoplane (**Figure 5**), which indicates that the rhizoplane is the most active compartment of the plant root. In addition, our findings demonstrated that the modularity of the rhizoplane bacterial and fungal networks was higher than that of the rhizosphere (**Table S5**). Since modules can be interpreted as microbial niches (Eiler et al., 2012; Zhao et al., 2020; Ling et al., 2022), a higher modularity of the rhizoplane suggests that niche differentiation is more pronounced in the rhizoplane. The rhizoplane network allocates more modules for more executive functions, partly reflecting the rapid element cycling in the rhizoplane (Ling et al., 2022). A higher modularity the of rhizoplane further confirmed that the rhizoplane is the most active compartment of the plant root. More enriched bacterial and fungal OTUs were observed in the rhizoplane (**Figures 2**, **3** and **Table S3**) indicating that the rhizoplane is a hotspot for plant–microbe–environment interactions (Xiong et al., 2021).

Our results further demonstrated that the bacterial co-occurrence network in the rhizoplane was more complex than that in the rhizosphere. This is understandable because plant roots in the rhizoplane regulate the soil environment by releasing large quantities of exudates, which may enhance microbial interactions (Zhalnina et al., 2018). Negative correlations may be a result of abiotic variation (niche heterogeneity) in the environment (Götzenberger et al., 2012; Brazeau and Schamp, 2019). A microbial community with a large proportion of negative correlations is thought to be stable (Coyte et al., 2015); a lower ratio of the negative-to-positive edges of the bacterial network in the rhizoplane indicates that the network is less stable in the rhizoplane than in the rhizosphere and soil (**Table S5**). Our observation is similar to a previous study that the microbial community in the rhizosphere is less stable than in soil (Ling et al., 2022). This is mainly because the rhizoplane is characterized by very high temporal dynamics compared to the more static conditions in the soil (Herron et al., 2013; Kuzyakov and Razavi, 2019; Munoz-Ucros et al., 2021). In contrast to the rhizoplane, the networks of the soil and rhizosphere were more stable. Our results differed slightly from a previous study conducted in switch grass that demonstrated that soil networks harbor more negative associations compared to the rhizosphere networks (Ceja-Navarro et al., 2021). The difference can be caused by the different classification of rhizo-ompartments as the previous study did not distinguish the rhizoplane compartment.

### The assembly of bacterial and fungal communities were mainly driven by plant-specific factors and environment variables.

Our results were in line with previous studies demonstrating that soils are generally enriched by dominant phyla Acidobacteria (Fan et al., 2017; Ling et al., 2022) and Chloroflexi (Ling et al., 2022) (**Figure S3**), which are oligotrophs (Jones et al., 2009; Finn et al., 2017). The rhizoplane was inhabited by a greater number of Proteobacteria (**Figure S3**), which are copiotrophs (Ling et al., 2022) and fast-growing (Schöps et al., 2018). We also found that Acidobacteria, which decreased monotonically with increasing soil pH (Bryant et al., 2008), were depleted in the rhizoplane (**Figure S3**). Actually, roots can regulate soil pH and change the buffering capacity of the rhizosphere soil (Wang et al., 2011; Mommer et al., 2016). For fungal composition, the relative abundance of taxa related to arbuscular mycorrhiza (AM) was the highest in root endosphere. AM symbiosis is formed by a monophyletic group of fungi from the phylum Glomeromycota, which can form AM symbiosis with 70%–90% of land plant roots (Parniske, 2008). We also observed that the enriched fungal OTU belongs to class Sordariomycetes and Dothideomycetes, which was consistent with a previous study (Durán et al., 2018). In addition, the phylogenetic tree revealed that core OTUs significantly enriched or depleted in the rhizoplane exhibited relatively close phylogenetic distances, which provides an evidence of plant rhizoplane convergent selection (Thiergart et al., 2020; He et al., 2022).

The microbial community in the root endosphere is mainly affected by plant genetic factors (Edwards et al., 2015; Fitzpatrick et al., 2018). A previous study suggested that the distribution of bacterial phyla inside the plant roots might be similar for all plants (Edwards et al., 2015). In our study, Ascomycota was enriched in the root endosphere (**Figure S3**), while Basidiomycota was depleted, which was not consistent with Zhuang et al. (2020) study on mangrove root–associated microbiomes but was in line with a study conducted in the oil palm root (Kirkman et al., 2022). This suggests that, unlike root endosphere bacteria, which are less affected by plant genetics (Edwards et al., 2015), endosphere fungi are more affected by plant genetic factors. Root endosphere bacterial phylum composition has more associations with environment variables (**Figure S12**), while fungal composition has less associations (**Figure S13**). This further indicates that endosphere bacteria were more affected by environmental variables, while endosphere fungi are more affected by plant genetic factors.

There is a general belief that different assembly rules control the establishment of microbial communities in the rhizosphere, rhizoplane, and endosphere of plants (Attia et al., 2022). Roots usually secrete a lot of organic compounds into the rhizosphere (Bais et al., 2006; Ling et al., 2022), which acts as a driving force for microbial growth and activity (Loeppmann et al., 2016; Ma et al., 2018). In our study, we identified that the stochastic process dominates bacterial community assemblages in the soil, rhizosphere, and rhizoplane (**Figure 7**). These findings were not consistent with Fan et al. (2017) and Zhuang et al. (2020) results that the deterministic process dominates root-associated bacterial community assembly. As stochastic processes increase with a rise in nutrient amounts (Chase, 2010; Zhou and Ding, 2017; Feng et al., 2018), we suggest that the microhabitats for these three compartments are nutrient-rich. In addition, rhizosphere is a relatively homogeneous environment (Li et al., 2021), which may be the possible reason for the more stochastic processes of the bacterial and fungal communities in the rhizoplane than in the rhizosphere and soil.

## Conclusion

This study provided fundamental insights into the root-associated microbial community, revealing plant–soil driven microbial composition, diversity, and assemblages of rubber trees. Our findings proposed a new view that microbes can disperse bidirectionally across different compartments of plant roots. In addition, our results suggest that the rhizoplane is the hotspot of interactions between plants and microorganisms, and the microbial community in the rhizoplane, closely related nutrient cycling, is more complex but less stable. These results expand our understanding of the microbial community structure, diversity, and assembly from the soil to the root endosphere of a plant, which may provide the scientific basis for sustainable agriculture through biological process management.

## Data availability statement

The datasets presented in this study can be found in online repositories. The names of the repository/repositories and accession number(s) can be found in the article/**Supplementary Material**.

## Author contributions

GL: conceptualization, methodology, and writing. YL: reviewing and editing. WY and ZW: investigation. All authors contributed to the article and approved the submitted version.
