# Supplementary material for: Diversity and assembly of root-associated microbiomes of rubber trees
Source: Front Plant Sci. 2023 Mar 31;14:1136418. doi: 10.3389/fpls.2023.1136418 (PMC10102524; doi:10.3389/fpls.2023.1136418)
Supplement: Supplementary file 1 [file DataSheet_1.docx]

**Supplementary materials**

**Diversity and assembly of root-associated microbiomes of rubber trees**

Lan Guoyu^1,2*^, Wei Yaqing^1,3+^ , Li Yuwu^4+^, Wu Zhixiang^1,2^

1. Rubber Research Institute, Chinese Academy of Tropical Agricultural Sciences, Haikou, Hainan 571101, P.R. China

2. Hainan Danzhou Tropical Agro-ecosystem National Observation and Research Station, Danzhou, Hainan 571737, P.R. China

3. College of Ecology and Environment, Hainan University, Haikou, Hainan 570228, P.R. China 4. 4. College of Landscape Architecture and Forestry,Qingdao Agricultural University, No. 700, Changcheng Road, Chengyang District, Qingdao, Shandong Province 266109, P.R. China

*Corresponding author: Lan Guoyu and Li Yuwu

Tel: +86-898-23301800

Fax: +86-898-23300315

E-mail: [langyrri@163.com](mailto:langyrri@163.com) & [liyuwu@qau.edu.cn](mailto:liyuwu@qau.edu.cn)

**Supplementary materials**

**Figure S1** Sampling sites (18 sampling sites in total) of root-associated microbiome for rubber tree root in south China. We selected three plots in each site, and sampled in both dry season and rainy season, thus we got a total of 144 samples for each compartment.

**Figure S2** Sampling design of soil (A) and rubber tree root (B). Blue solid circle is the location for soil sampling, red solid circle is the location for rubber tree root and rhizosphere soil, while the larger soil brown circle is the trunk of the rubber tree.

**Figure S3** The bar plot showing significant difference in bacterial and fungal community compositions at phylum level among different compartments of rubber tree root. * *p* < 0.05, ** *p* < 0.01, *** *p* < 0.001

**Figure S4** Beta-diversity of root-associated bacterial and fungal communities of different compartments of rubber tree root. A: PCoA ordinations based on the Bray-Curtis similarity of bacterial and fungal community beta-diversity of four compartments of root for rubber trees. Each solid circle represents an individual sample. B: Boxplots illustrate the distance to the centroid for compartments in the PCoA.

**Figure S5** The bar plot showing the abundance and percentage of core bacterial and fungal community composition at phylum level across different compartments of rubber tree root. Core bacterial OTUs: present in all samples of each compartment; and with a relative abundance >0.01%. Core fungal OTUs, present in at least 60 % samples of each compartment and with a relative abundance ≥ 0.01%.

**Figure S6** The bar plot showing significant difference in core bacterial and fungal community composition in each phylum arcorss different compartments of rubber tree root. Core bacterial OTUs: present in all samples of each compartment; and with a relative abundance >0.01%. Core fungal OTUs, present in at least 60 % samples of each compartment and with a relative abundance ≥ 0.01%.* *p* < 0.05, ** *p* < 0.01, *** *p* < 0.001.

**Figure S7** The enrichment and depletion patterns of the root-associated bacterial and fungal microbiomes in each phylum in each compartment compared with soil. The y-axis represents the fold-change (FC) in abundance compared with soil, and the x-axis represents the logarithm (base10) of abundance for each phylum. Numbers on the right of the panel indicate enriched or depleted OTUs. Blue solid circles indicate endosphere, dark green solid circles indicate rhizoplane, while light blue solid circles indicate rhizosphere.

**Figure S8**: The bar plot showing the relative abundance fungal groups as inferred by FUNguild for different compartments of rubber tree root.

**Figure S9** Network of root-associated core bacterial and fungal communities of different compartments of rubber tree root. Green solid circles represent bacteria, red solid circles represent fungi; green solid lines represent positive correlations; red solid lines represent negative correlations.

**Figure S10** Number of shared and unique edges of soil bacterial and fungal networks in different compartments. The number where the two circles cross is number of shared edges.

**Figure S11:** Network degree of core bacterial and fungal communities for different compartments of rubber tree root.

**Figure S12** Heat maps displaying pearson correlation coefficients between the environmental variables and relative abundance of bacterial phylum of different compartments of rubber tree root. SOM: soil organic matter; TN: total nitrogen; TP: total phosphorus; TK: total potassium; WC: water content; pH: soil pH. * *p* < 0.05, ** *p* < 0.01, *** *p* < 0.001

**Figure S13** Heat maps displaying pearson correlation coefficients between the environmental variables and relative abundance of fungal phylum of different compartments of rubber tree root. SOM: soil organic matter; TN: total nitrogen; TP: total phosphorus; TK: total potassium; WC: water content; pH: soil pH. * *p* < 0.05, ** *p* < 0.01, *** *p* < 0.001

**Table S1** Information on sampling sites of root-associated microbiome for rubber tree root in south of China.

**Table S2** Analysis of similarities (ANOSIM) of the bacterial and fungal community composition among the four compartments based on Bray-Curtis distance metric

**Table S3** The core enriched and depleted bacterial and fungal OTUs of in each compartment compared with soil.

**Table S4** The potential sources of rubber tree root associated bacterial and fungal communities of different compartments estimated by FEAST.

**Table S5** The bacterial and fungal community network edges and modularity of different compartments of rubber tree root

**Table S6** The core bacterial and fungal community network edges and modularity of different compartments of rubber tree root

**
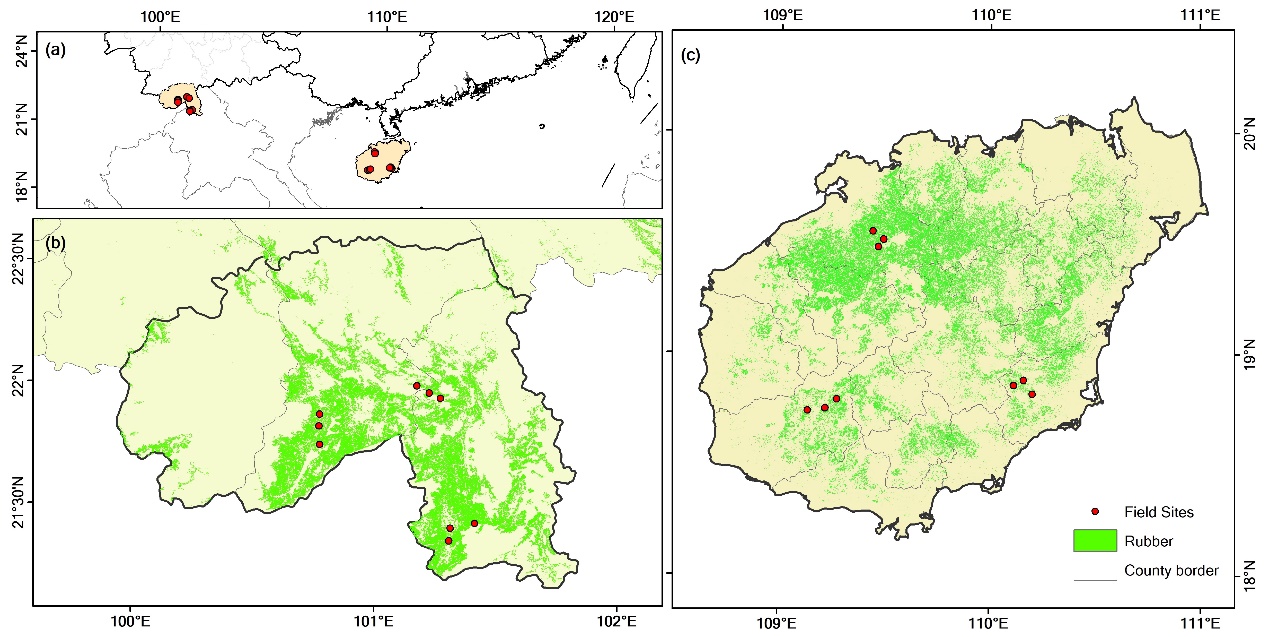
**

**Figure S1** Sampling sites (18 sampling sites in total) of root-associated microbiome for rubber tree root in south China. We selected three plots in each site, and sampled in both dry season and rainy season, thus we got a total of 144 samples for each compartment.


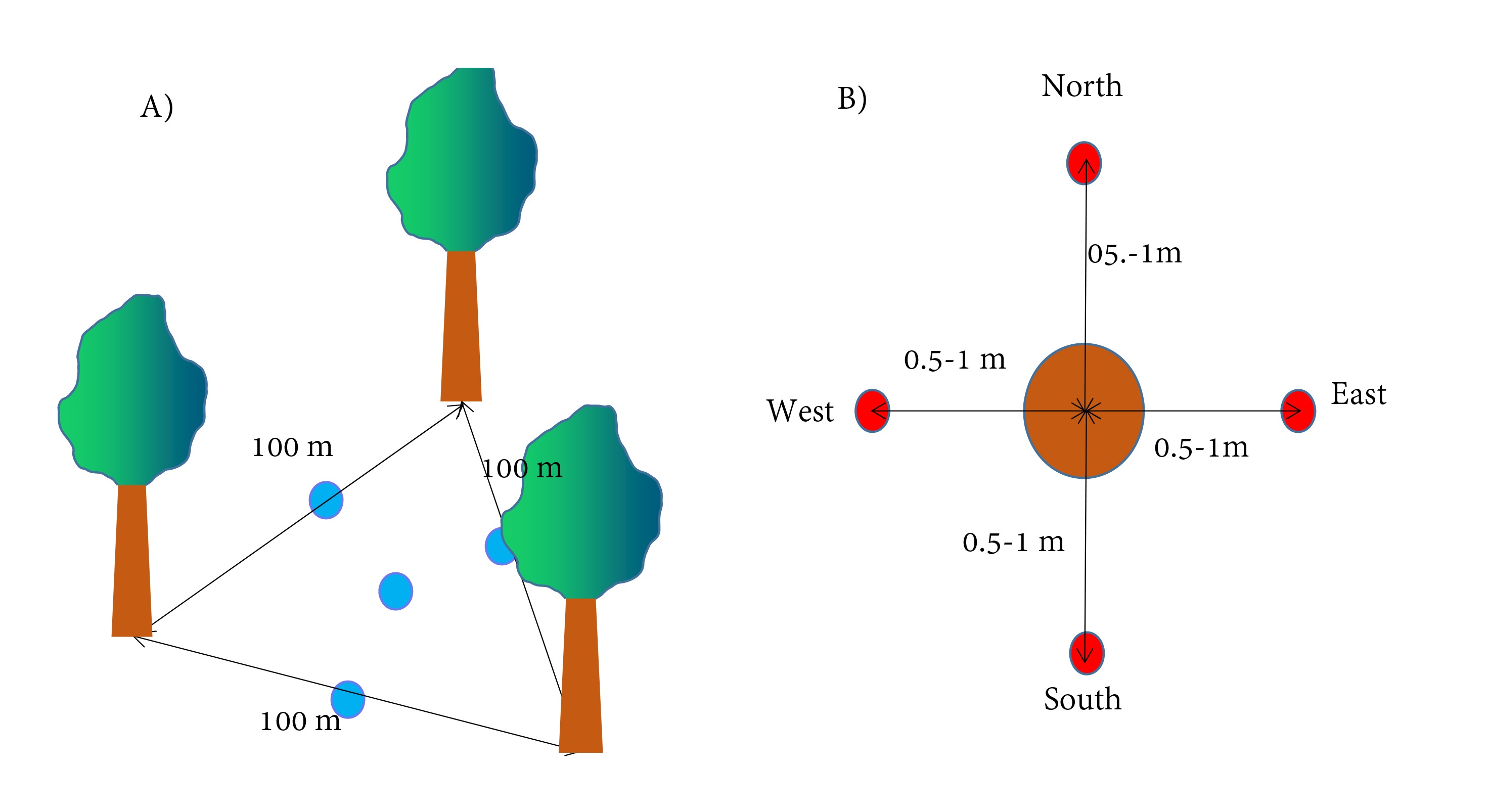


**Figure S2** Sampling design of soil (A) and rubber tree root (B). Blue solid circle is the location for soil sampling, red solid circle is the location for rubber tree root and rhizosphere soil, while the larger soil brown circle is the trunk of the rubber tree.


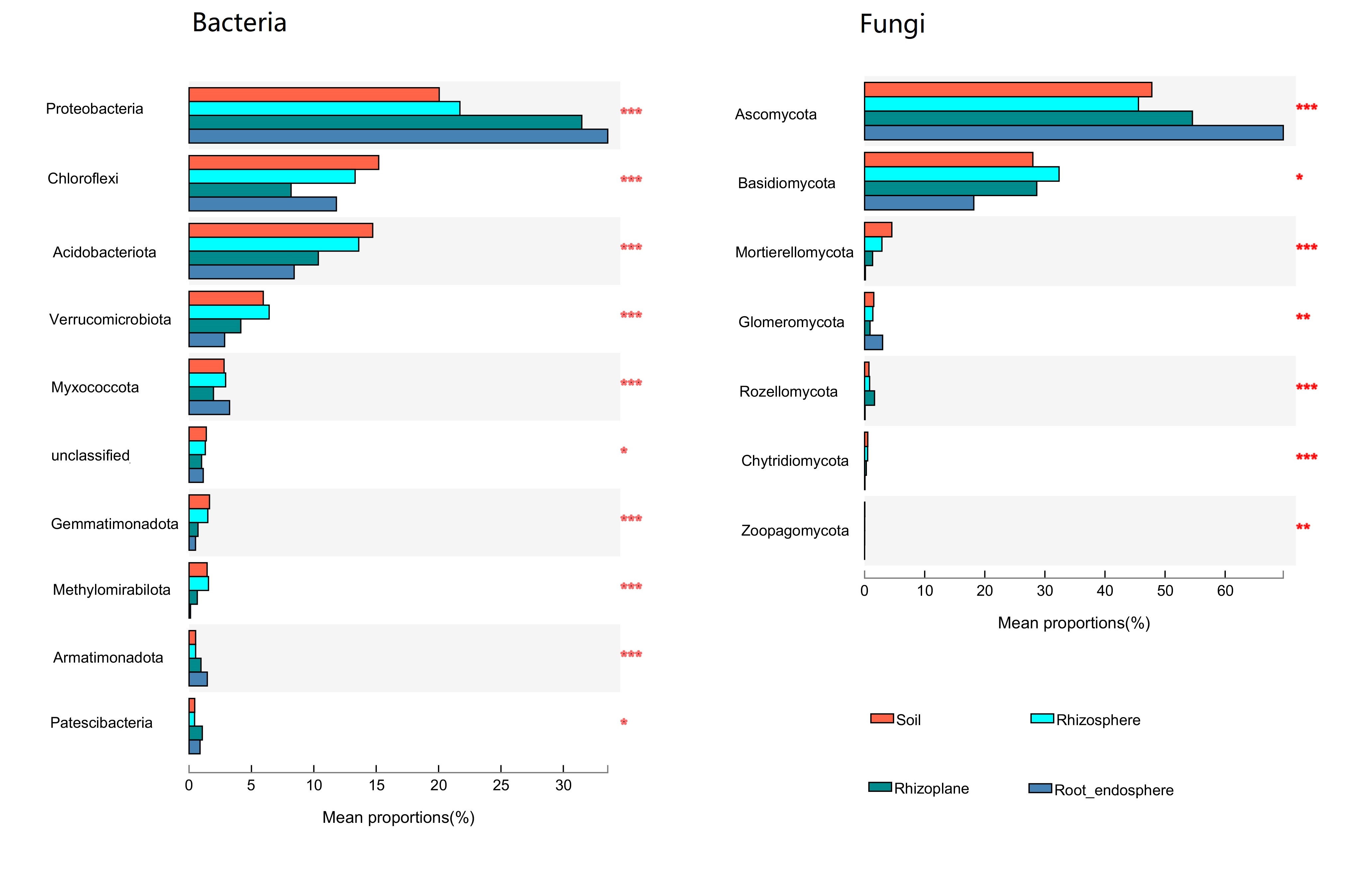


**Figure S3** The bar plot showing significant difference in bacterial and fungal community compositions at phylum level among different compartments of rubber tree root. * *p* < 0.05, ** *p* < 0.01, *** *p* < 0.001


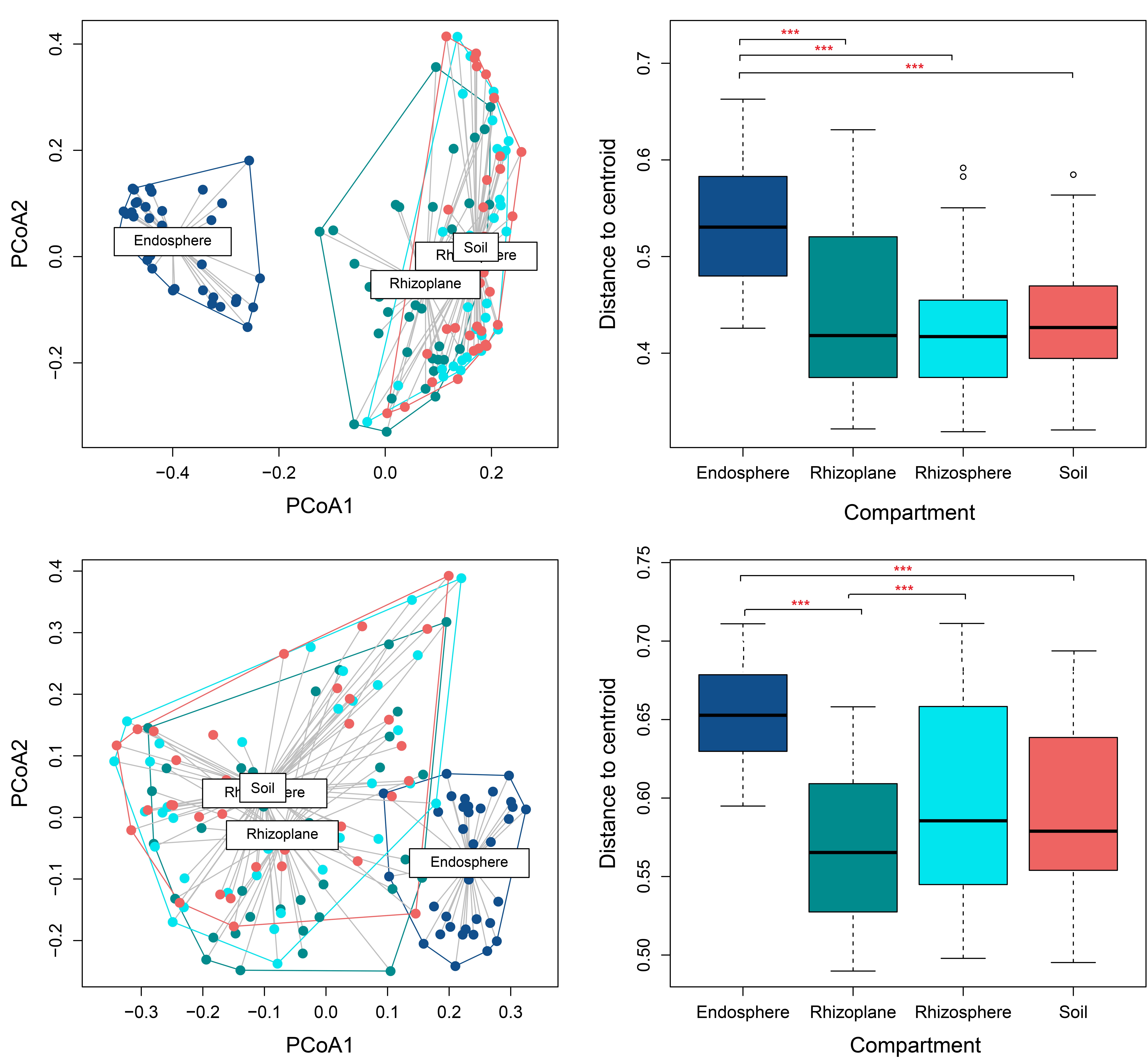


**Figure S4** Beta-diversity of root-associated bacterial and fungal communities of different compartments of rubber tree root. A: PCoA ordinations based on the Bray-Curtis similarity of bacterial and fungal community beta-diversity of four compartments of root for rubber trees. Each solid circle represents an individual sample. B: Boxplots illustrate the distance to the centroid for compartments in the PCoA. * *p* < 0.05, ** *p* < 0.01, *** *p* < 0.001.


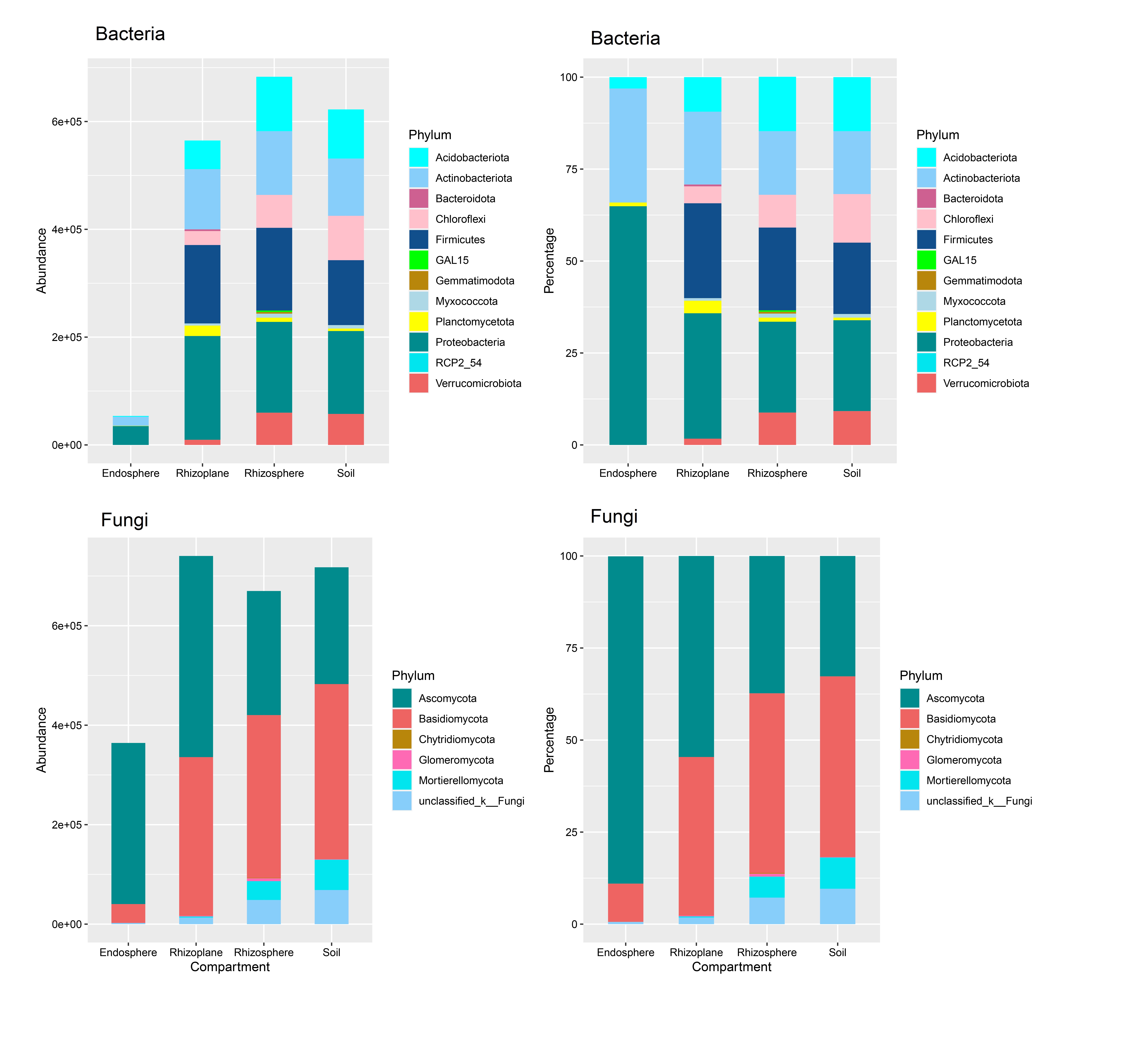


**Figure S5** The bar plot showing the abundance and percentage of core bacterial and fungal community composition at phylum level across different compartments of rubber tree root. Core bacterial OTUs: present in all samples of each compartment; and with a relative abundance >0.01%. Core fungal OTUs, present in at least 60 % samples of each compartment and with a relative abundance ≥ 0.01%.


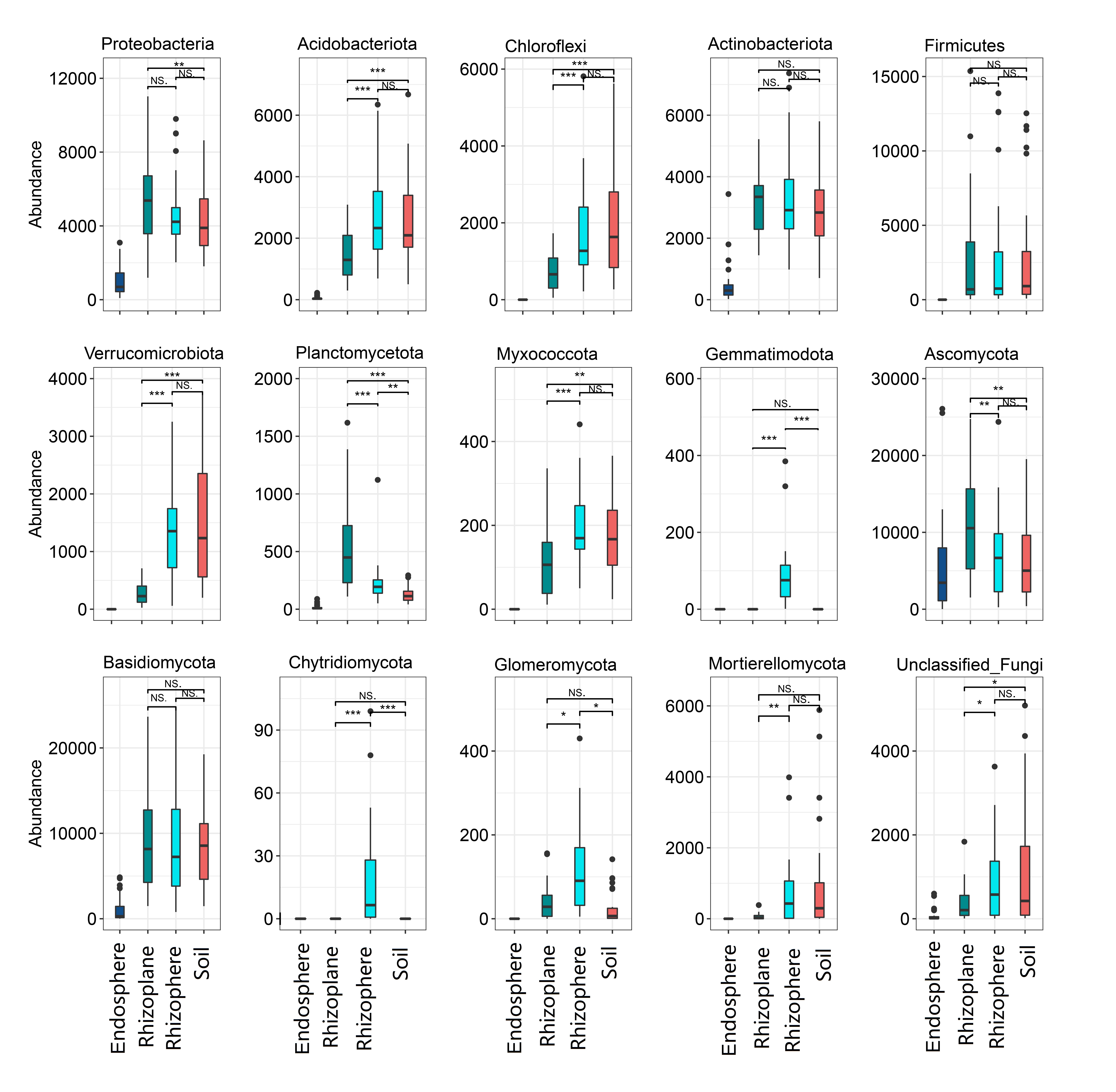


**Figure S6** The bar plot showing significant difference in core bacterial and fungal community composition in each phylum among different compartments of rubber tree root. * *p* < 0.05, ** *p* < 0.01, *** *p* < 0.001. Core bacterial OTUs: present in all samples of each compartment; and with a relative abundance >0.01%. Core fungal OTUs, present in at least 60 % samples of each compartment and with a relative abundance ≥ 0.01%.


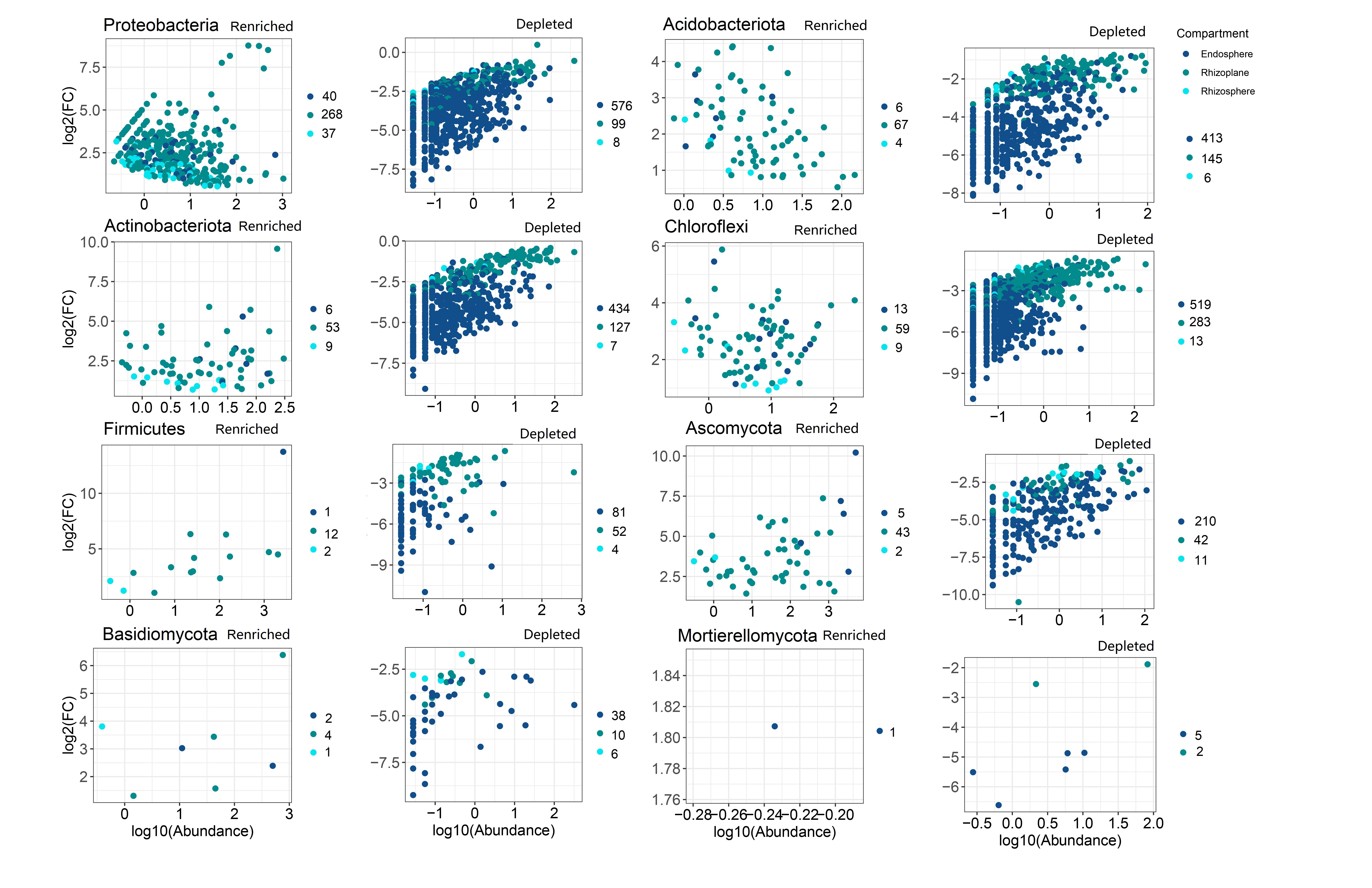
 **Figure S7** The enrichment and depletion patterns of the root-associated bacterial and fungal microbiomes in each phylum in each compartment compared with soil. The y-axis represents the fold-change (FC) in abundance compared with soil, and the x-axis represents the logarithm (base10) of abundance for each phylum. Numbers on the right of the panel indicate enriched or depleted OTUs. Blue solid circles indicate endosphere, dark green solid circles indicate rhizoplane, while light blue solid circles indicate rhizosphere.


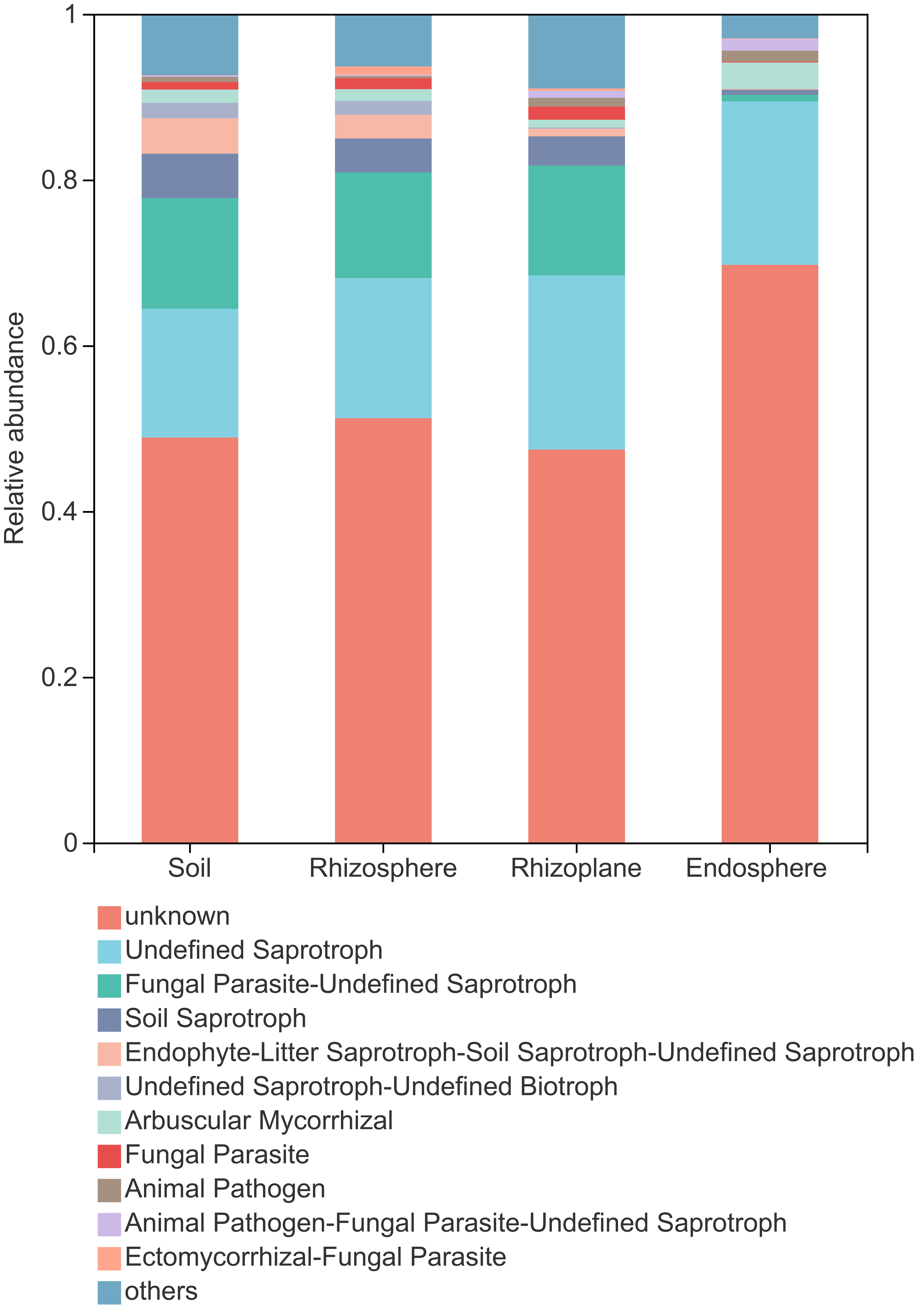


**Figure S8**: The barplot showing the relative abundance fungal groups as inferred by FUNguild for different compartments of rubber tree root.


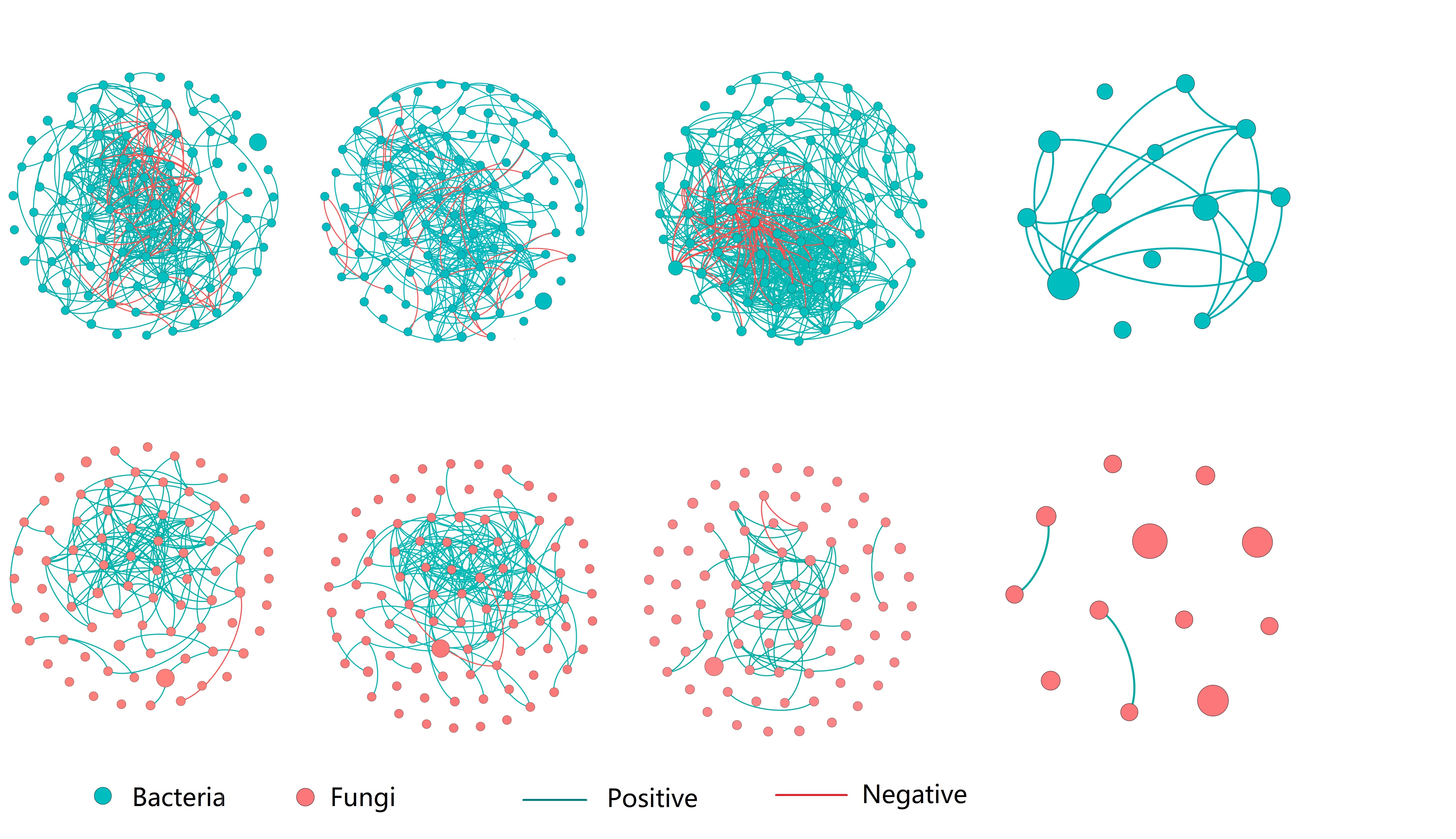


**Figure S9** Network of root-associated core bacterial and fungal communities of different compartments of rubber tree root. Green solid circles represent bacteria, red solid circles represent fungi; green solid lines represent positive correlations; red solid lines represent negative correlations.


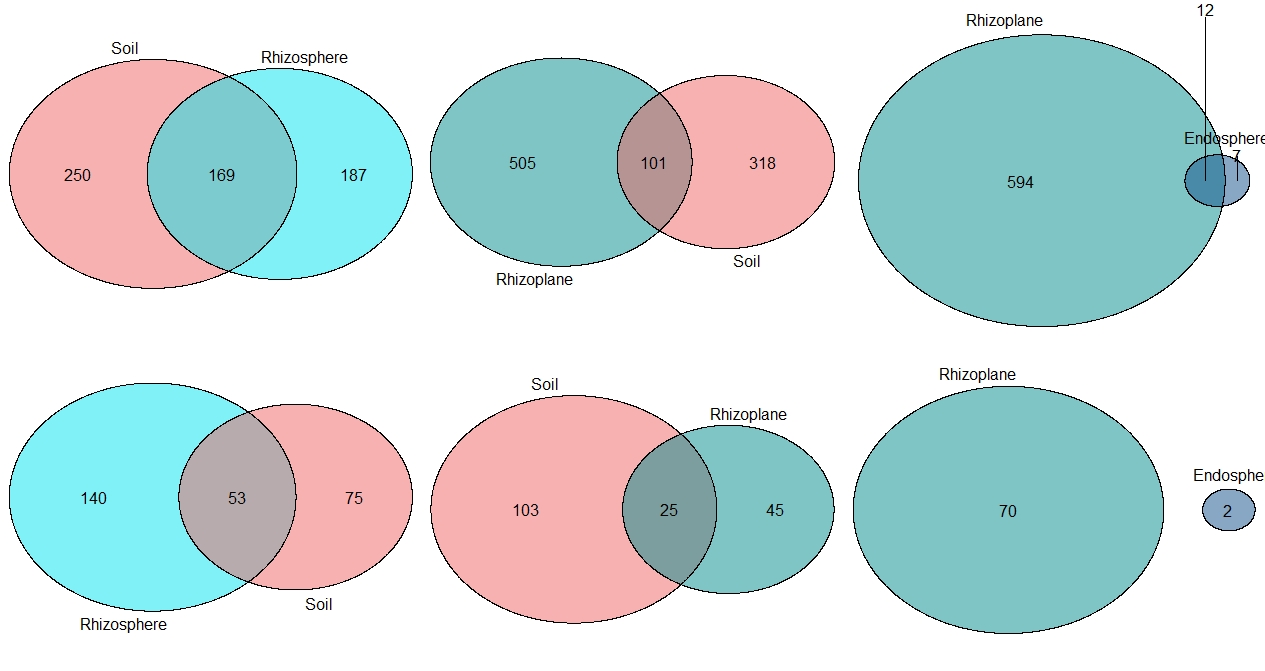


**Figure S10** Number of shared and unique edges of soil bacterial and fungal networks in different compartments. The number where the two circles cross is number of shared edges.


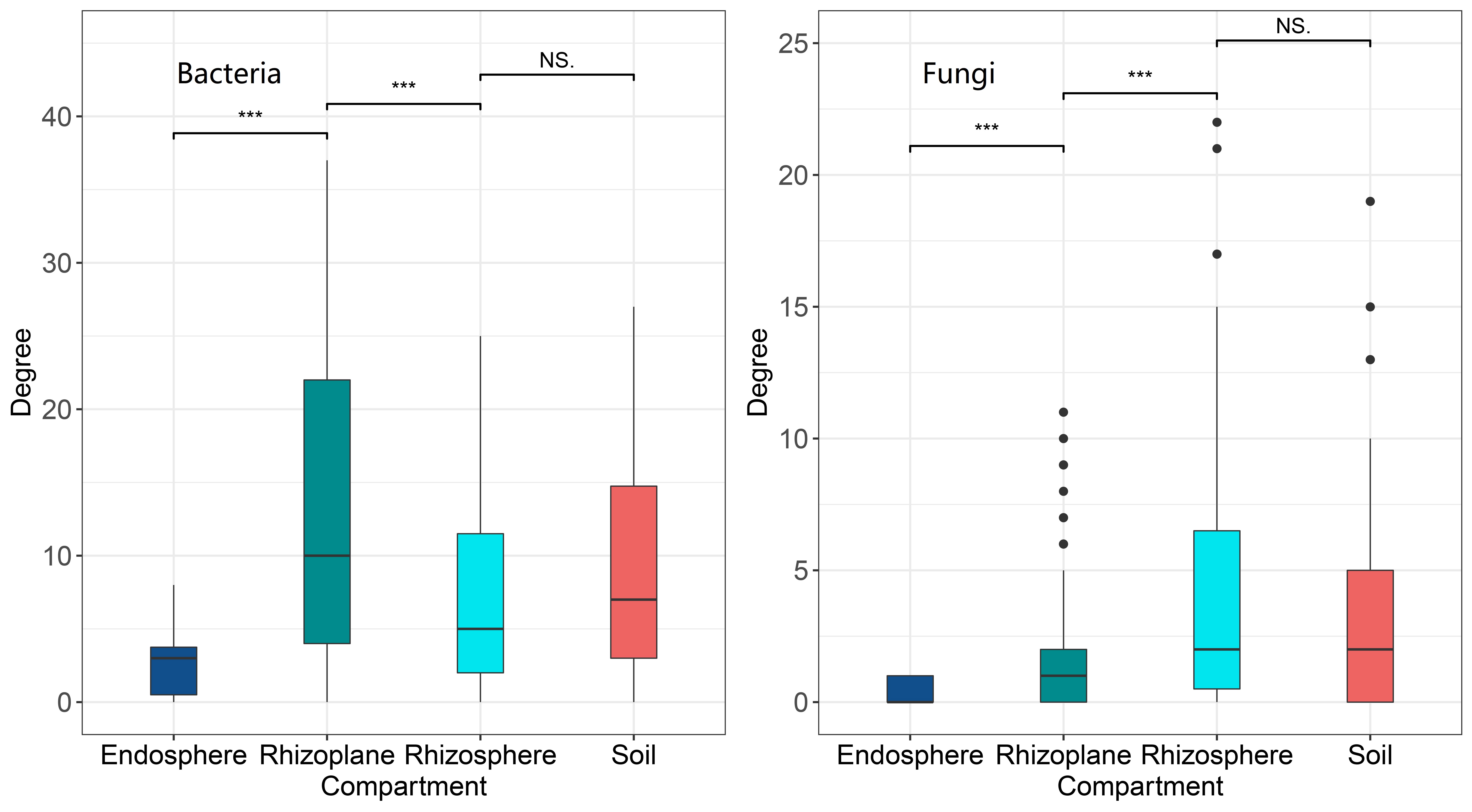


**Figure S11:** Network degree of core bacterial and fungal communities for different compartments of rubber tree root. * *p* < 0.05, ** *p* < 0.01, *** *p* < 0.001

**
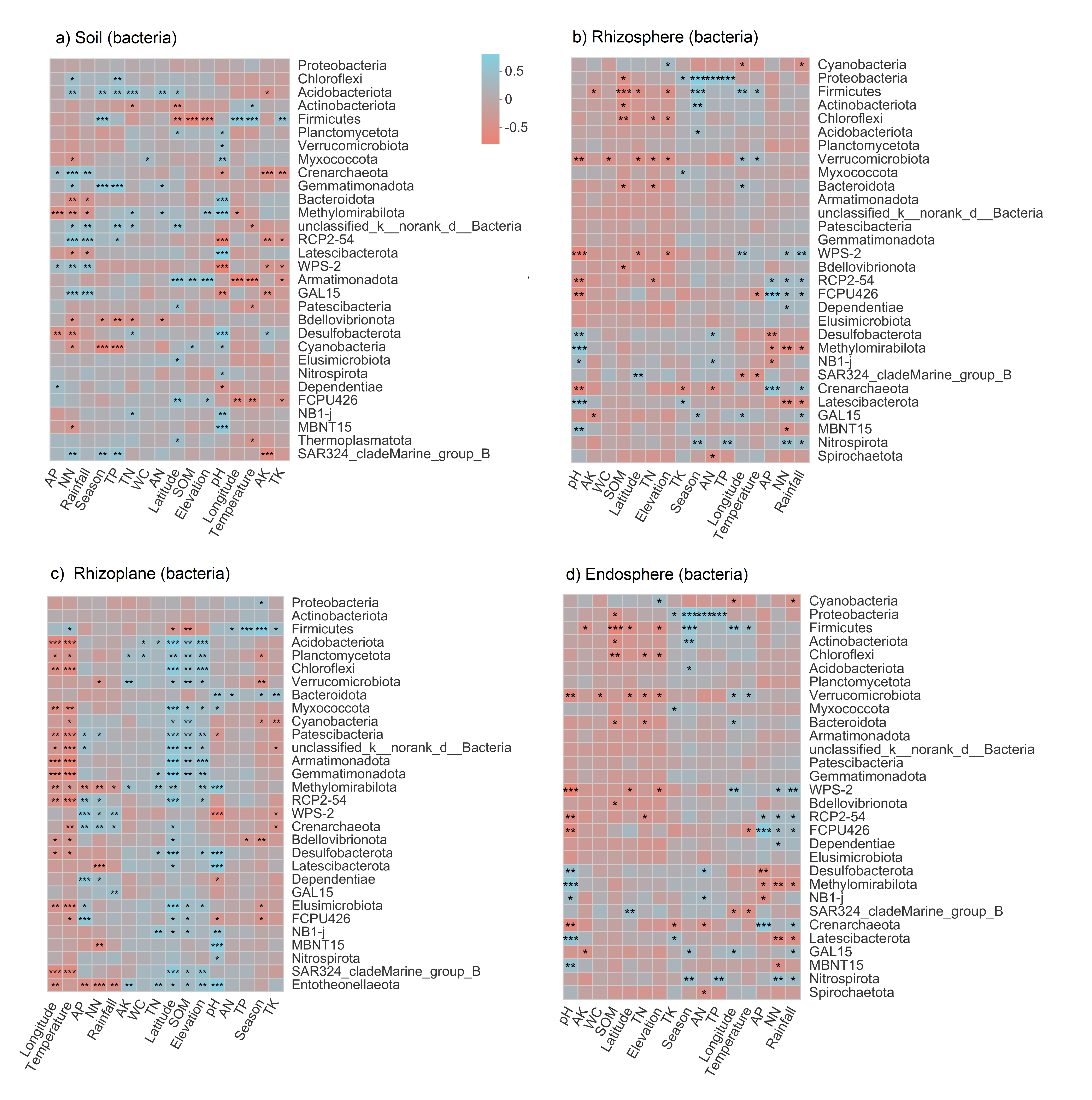
**

**Figure S12** Heat maps displaying pearson correlation coefficients between the environmental variables and relative abundance of bacterial phylum of different compartments of rubber tree root. SOM: soil organic matter; TN: total nitrogen; TP: total phosphorus; TK: total potassium; WC: water content; pH: soil pH. * *p* < 0.05, ** *p* < 0.01, *** *p* < 0.001


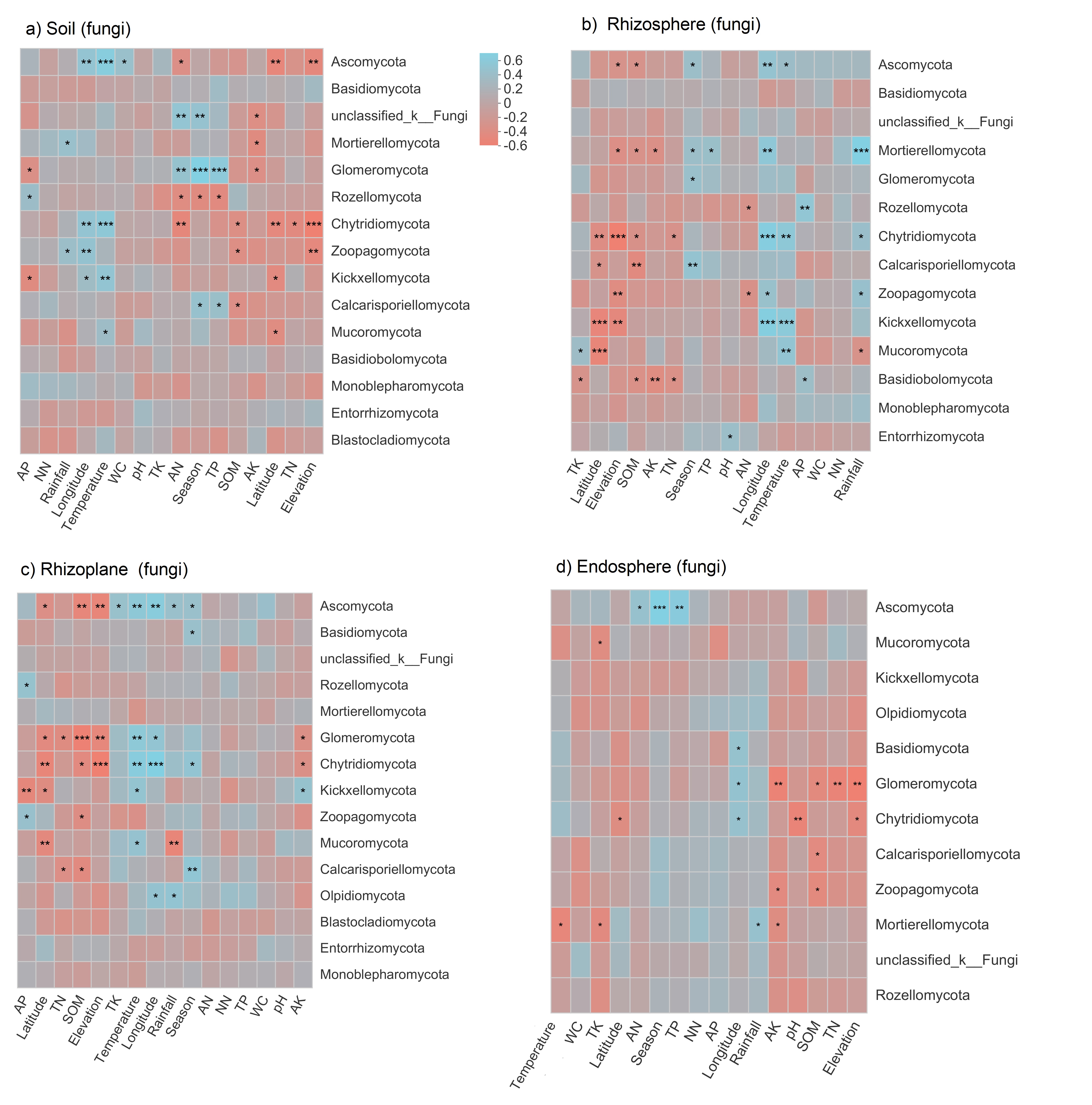


**Figure S13** Heat maps displaying pearson correlation coefficients between the environmental variables and relative abundance of fungal phylum of different compartments of rubber tree root. SOM: soil organic matter; TN: total nitrogen; TP: total phosphorus; TK: total potassium; WC: water content; pH: soil pH. * *p* < 0.05, ** *p* < 0.01, *** *p* < 0.001

**Table S1** Information on sampling sites of root-associated microbiome for rubber tree root in south of China

| **Plot** | **Site** | **Longitude** | **Latitude** | **Rainfall**  **（mm）** | **Temperature**  **（℃）** | **Elevation（m）** | |  |
| --- | --- | --- | --- | --- | --- | --- | --- | --- |
| 1 | Danzhou | 109° 29'27.4" | 19° 31'09" | 1856.02 | 23.67 | 134 |  | |
| 2 | Danzhou | 109° 26'25" | 19° 33'19.6" | 1856.02 | 23.67 | 110 |  | |
| 3 | Danzhou | 109° 28'1.8" | 19° 29'5.1" | 1856.02 | 23.67 | 159 |  | |
| 4 | Wanning | 110° 12'7.9" | 18° 49'19.3" | 1858.83 | 24.73 | 162 |  | |
| 5 | Wanning | 110° 9'36.2" | 18° 53'3.0" | 1858.83 | 24.73 | 166 |  | |
| 6 | Wanning | 110° 6'47.5" | 18° 51'38.6" | 1858.83 | 24.73 | 160 |  | |
| 7 | Ledong | 109°8'9.6" | 18° 44'35.2" | 1189.12 | 25.29 | 165 |  | |
| 8 | Ledong | 109° 13'9" | 18°45'16.6" | 1189.12 | 25.29 | 174 |  | |
| 9 | Ledong | 109° 16'27.1" | 18° 47'44.6" | 1189.12 | 25.29 | 173 |  | |
| 10 | Jinghong | 100° 46'44.6" | 21° 51'40.8" | 1513.75 | 21.58 | 651 |  | |
| 11 | Jinghong | 100° 46'31.4" | 21° 48'45.3" | 1513.75 | 21.58 | 630 |  | |
| 12 | Jinghong | 100° 46'44.8" | 21°44'12.6" | 1513.75 | 21.58 | 635 |  | |
| 13 | Menglun | 101° 13'46.8" | 21°56'52.9" | 1513.75 | 21.58 | 618 |  | |
| 14 | Menglun | 101° 10'42.1" | 21°58'39.7" | 1513.75 | 21.58 | 1069 |  | |
| 15 | Menglun | 101°16'28.5" | 21°55'34.2" | 1513.75 | 21.58 | 549 |  | |
| 16 | Mengpeng | 101°24'58.1" | 21°24'39.9" | 1513.75 | 21.58 | 619 |  | |
| 17 | Mengpeng | 101°18'56" | 21°23'33.2" | 1513.75 | 21.58 | 600 |  | |
| 18 | Mengpeng | 101°18'30.6" | 21°20'26.8" | 1513.75 | 21.58 | 615 |  | |

**Table S2** Analysis of similarities (ANOSIM) of the bacterial and fungal community composition among the four compartments based on Bray-Curtis distance metric

| **Group** | **Bacteria** | | **Fungi** | |
| --- | --- | --- | --- | --- |
|  | ***R*-statistics** | ***P-v*alue** | ***R*-statistics** | ***P-v*alue** |
| Soil-rhizosphere | 0.0081 | 0.792 | 0.0226 | 0.987 |
| Soil-rhizoplane | 0.2549 | 0.001 | 0.1105 | 0.001 |
| Soil-endosphere | **0.9996** | **0.001** | **0.5855** | **0.001** |
| Rhizosphere-rhizoplane | 0.2267 | 0.001 | 0.0755 | 0.002 |
| Rhizospere-endosphere | **0.9993** | **0.001** | **0.5150** | **0.001** |
| Rhizoplane-endosphere | **0.9852** | **0.001** | **0.3096** | **0.001** |

**Table S3** The core enriched and depleted bacterial and fungal OTUs of in each compartment compared with soil.

| **ID** | **Abundance** | **FC** | **p-value** | **Class** | **Phylum** | **Compartment** | **Enrich/Depleted** | **Taxon** |
| --- | --- | --- | --- | --- | --- | --- | --- | --- |
| OTU15880 | 20 | 0.03 | 0 | Alphaproteobacteria | Proteobacteria | Endosphere | Depleted | Bacteria |
| OTU15005 | 93 | 0.12 | 0 | Alphaproteobacteria | Proteobacteria | Endosphere | Depleted | Bacteria |
| OTU10109 | 24 | 0.12 | 0 | Actinobacteria | Actinobacteriota | Endosphere | Depleted | Bacteria |
| OTU15123 | 71 | 0.14 | 0 | Actinobacteria | Actinobacteriota | Endosphere | Depleted | Bacteria |
| OTU17119 | 91 | 0.5 | 0.0035 | Alphaproteobacteria | Proteobacteria | Endosphere | Depleted | Bacteria |
| OTU12092 | 46 | 0.58 | 0.0411 | Acidobacteriae | Acidobacteriota | Endosphere | Depleted | Bacteria |
| OTU15285 | 129 | 0.13 | 0.0002 | AD3 | Chloroflexi | Rhizoplane | Depleted | Bacteria |
| OTU15078 | 56 | 0.14 | 0.0009 | AD3 | Chloroflexi | Rhizoplane | Depleted | Bacteria |
| OTU11222 | 638 | 0.22 | 0.003 | Bacilli | Firmicutes | Rhizoplane | Depleted | Bacteria |
| OTU25337 | 85 | 0.34 | 0 | Acidobacteriae | Acidobacteriota | Rhizoplane | Depleted | Bacteria |
| OTU10109 | 79 | 0.41 | 0.0001 | Actinobacteria | Actinobacteriota | Rhizoplane | Depleted | Bacteria |
| OTU14878 | 114 | 0.44 | 0.0003 | Actinobacteria | Actinobacteriota | Rhizoplane | Depleted | Bacteria |
| OTU8703 | 91 | 0.45 | 0.0001 | Acidobacteriae | Acidobacteriota | Rhizoplane | Depleted | Bacteria |
| OTU8927 | 89 | 0.45 | 0.0229 | Verrucomicrobiae | Verrucomicrobiota | Rhizoplane | Depleted | Bacteria |
| OTU12917 | 177 | 0.47 | 0.0058 | TK10 | Chloroflexi | Rhizoplane | Depleted | Bacteria |
| OTU24572 | 57 | 0.6 | 0.0066 | Thermoleophilia | Actinobacteriota | Rhizoplane | Depleted | Bacteria |
| OTU22408 | 64 | 0.61 | 0.0329 | Actinobacteria | Actinobacteriota | Rhizoplane | Depleted | Bacteria |
| OTU15123 | 310 | 0.63 | 0.0395 | Actinobacteria | Actinobacteriota | Rhizoplane | Depleted | Bacteria |
| OTU25910 | 75 | 0.65 | 0.0393 | Thermoleophilia | Actinobacteriota | Rhizoplane | Depleted | Bacteria |
| OTU23596 | 62 | 0.66 | 0.0043 | Thermoleophilia | Actinobacteriota | Rhizoplane | Depleted | Bacteria |
| OTU12107 | 111 | 0.67 | 0.0442 | Verrucomicrobiae | Verrucomicrobiota | Rhizoplane | Depleted | Bacteria |
| OTU15880 | 396 | 0.69 | 0.0451 | Alphaproteobacteria | Proteobacteria | Rhizoplane | Depleted | Bacteria |
| OTU16555 | 78 | 0.75 | 0.0451 | Thermoleophilia | Actinobacteriota | Rhizoplane | Depleted | Bacteria |
| OTU19352 | 88 | 1.45 | 0.0308 | Acidobacteriae | Acidobacteriota | Rhizoplane | Enriched | Bacteria |
| OTU14337 | 104 | 1.77 | 0.0166 | Acidobacteriae | Acidobacteriota | Rhizoplane | Enriched | Bacteria |
| OTU11019 | 197 | 1.81 | 0.0105 | Alphaproteobacteria | Proteobacteria | Rhizoplane | Enriched | Bacteria |
| OTU12092 | 146 | 1.83 | 0.0065 | Acidobacteriae | Acidobacteriota | Rhizoplane | Enriched | Bacteria |
| OTU22472 | 115 | 1.89 | 0.006 | Gammaproteobacteria | Proteobacteria | Rhizoplane | Enriched | Bacteria |
| OTU25900 | 139 | 1.9 | 0.0002 | Planctomycetes | Planctomycetota | Rhizoplane | Enriched | Bacteria |
| OTU11184 | 122 | 1.96 | 0 | Actinobacteria | Actinobacteriota | Rhizoplane | Enriched | Bacteria |
| OTU11097 | 1046 | 2 | 0 | Alphaproteobacteria | Proteobacteria | Rhizoplane | Enriched | Bacteria |
| OTU17119 | 426 | 2.33 | 0.0002 | Alphaproteobacteria | Proteobacteria | Rhizoplane | Enriched | Bacteria |
| OTU7574 | 185 | 2.34 | 0.0006 | Actinobacteria | Actinobacteriota | Rhizoplane | Enriched | Bacteria |
| OTU6741 | 439 | 2.47 | 0 | Gammaproteobacteria | Proteobacteria | Rhizoplane | Enriched | Bacteria |
| OTU9031 | 2027 | 22.81 | 0.0011 | Bacilli | Firmicutes | Rhizoplane | Enriched | Bacteria |
| OTU19166 | 321 | 0.05 | 0 | Tremellomycetes | Basidiomycota | Endosphere | Depleted | Fungi |
| OTU21161 | 56 | 0.1 | 0.001 | Sordariomycetes | Ascomycota | Endosphere | Depleted | Fungi |
| OTU14075 | 110 | 0.12 | 0.0001 | Sordariomycetes | Ascomycota | Endosphere | Depleted | Fungi |
| OTU10729 | 33 | 0.29 | 0.0233 | Dothideomycetes | Ascomycota | Endosphere | Depleted | Fungi |
| OTU541 | 496 | 5.26 | 0.025 | Tremellomycetes | Basidiomycota | Endosphere | Enriched | Fungi |
| OTU11507 | 3306 | 7 | 0.03 | Dothideomycetes | Ascomycota | Endosphere | Enriched | Fungi |
| OTU11930 | 2459 | 84.88 | 0.0376 | Sordariomycetes | Ascomycota | Endosphere | Enriched | Fungi |
| OTU19112 | 27 | 0.39 | 0.0165 | Sordariomycetes | Ascomycota | Rhizoplane | Depleted | Fungi |
| OTU10753 | 44 | 0.47 | 0.0251 | Sordariomycetes | Ascomycota | Rhizoplane | Depleted | Fungi |
| OTU11507 | 1408 | 2.98 | 0.0047 | Dothideomycetes | Ascomycota | Rhizoplane | Enriched | Fungi |
| OTU11578 | 220 | 3.64 | 0.0269 | Dothideomycetes | Ascomycota | Rhizoplane | Enriched | Fungi |
| OTU11761 | 152 | 9.95 | 0.0003 | Sordariomycetes | Ascomycota | Rhizoplane | Enriched | Fungi |
| OTU11536 | 505 | 36.11 | 0.0002 | Sordariomycetes | Ascomycota | Rhizoplane | Enriched | Fungi |
| OTU11930 | 1098 | 37.91 | 0.0068 | Sordariomycetes | Ascomycota | Rhizoplane | Enriched | Fungi |

**Table S4** The potential sources of rubber tree root associated bacterial and fungal communities of different compartments estimated by FEAST.

| **Taxon** | **Sink** | **Source1** | **Source2** | **Source3** | **Source4** |
| --- | --- | --- | --- | --- | --- |
| **Bacteria** | Soil | Rhizosphere  (50.05%) | Rhizoplane  (17.68%) | Endosphere  (4.35%) | Unknown  (27.92) |
|  | Rhizosphere | Soil  (48.03%) | Rhizoplane  (21.64%) | Endosphere  (5.84%) | Unknown  (24.49%) |
|  | Rhizoplane | Rhizosphere  (25.58%) | Endosphere  (18.69%) | Soil  （20.64%） | Unknown  （35.09%） |
|  | Endosphere | Rhizoplane  （36.28%） | Rhizosphere  （19.22%） | Soil  （7.32%） | Unknown  （37.18%） |
| **Fungi** | Soil | Rhizosphere  (70.60%) | Rhizoplane  (18.00%) | Endosphere  (1.50%) | Unknown  (9.90%) |
|  | Rhizosphere | Soil  (56.73%) | Rhizoplane  (35.50%) | Endosphere  (0.83%) | Unknown  (6.94%) |
|  | Rhizoplane | Rhizosphere  (50.60%) | Endosphere  (20.00%) | Soil  （20.60%） | Unknown  （8.80%） |
|  | Endosphere | Rhizoplane  （88.87%） | Rhizosphere  （2.50%） | Soil  （1.90%） | Unknown  （6.73%） |

**Table S5** The bacterial and fungal community network edges and modularity of different compartments of rubber tree root.

| **Taxon** | **Compartments** | **Total edges** | **Positive** | **Negative** | **Ratio (Negative/Positive)** | **Modularity** |
| --- | --- | --- | --- | --- | --- | --- |
| Bacteria | Soil | 10594 | 7267 | 3327 | 45.78 | 0.261 |
|  | Rhizosphere | 8609 | 5809 | 2800 | 48.20 | 0.344 |
|  | Rhizoplane | 10092 | 9017 | 1097 | 12.17 | 0.430 |
|  | Endosphere | 4727 | 4727 | 0 | 0 | 0.427 |
|  | Soil | 1508 | 1468 | 40 | 2.72% | 0.725 |
| Fungi | Rhizosphere | 1775 | 1660 | 115 | 6.93% | 0.615 |
|  | Rhizoplane | 1248 | 1186 | 62 | 5.23% | 0.695 |
|  | Endosphere | 1193 | 1192 | 1 | 0.08% | 0.792 |

**Table S6** The core bacterial and fungal community network edges and modularity of different compartments of rubber tree root.

| **Taxon** | **Compartments** | **Number of nodes** | **Total edges** | **Positive** | **Negative** | **Ratio (Negative/Positive)** | **Modularity** |
| --- | --- | --- | --- | --- | --- | --- | --- |
| Bacteria | Soil | 94 | 419 | 365 | 54 | 14.79 | 0.362 |
|  | Rhizosphere | 99 | 356 | 330 | 26 | 7.88 | 0.465 |
|  | Rhizoplane | 90 | 606 | 547 | 59 | 10.79 | 0.303 |
|  | Endosphere | 14 | 19 | 19 | 0 | 0 | 0.216 |
|  | Soil | 79 | 128 | 127 | 1 | 0.79 | 0.433 |
| Fungi | Rhizosphere | 91 | 193 | 191 | 2 | 1.04 | 0.329 |
|  | Rhizoplane | 77 | 71 | 69 | 2 | 2.90 | 0.473 |
|  | Endosphere | 12 | 2 | 2 | 0 | 0 | 0.500 |
